# Molecular Architecture of the Antiophidic Protein DM64 and its Binding Specificity to Myotoxin II From *Bothrops asper* Venom

**DOI:** 10.3389/fmolb.2021.787368

**Published:** 2022-01-27

**Authors:** Barbara S. Soares, Surza Lucia G. Rocha, Viviane A. Bastos, Diogo B. Lima, Paulo C. Carvalho, Fabio C. Gozzo, Borries Demeler, Tayler L. Williams, Janelle Arnold, Amy Henrickson, Thomas J. D. Jørgensen, Tatiana A. C. B. Souza, Jonas Perales, Richard H. Valente, Bruno Lomonte, Francisco Gomes-Neto, Ana Gisele C. Neves-Ferreira

**Affiliations:** ^1^ Laboratory of Toxinology, Oswaldo Cruz Institute, Rio de Janeiro, Brazil; ^2^ Department of Chemical Biology, Leibniz Forschungsinstitut für Molekulare Pharmakologie (FMP), Berlin, Germany; ^3^ Laboratory for Structural and Computational Proteomics, Carlos Chagas Institute, Curitiba, Brazil; ^4^ Dalton Mass Spectrometry Laboratory, University of Campinas, Campinas, Brazil; ^5^ Department of Biochemistry and Structural Biology, University of Texas Health Science Center at San Antonio, San Antonio, TX, United States; ^6^ Department of Chemistry and Biochemistry, University of Lethbridge, Lethbridge, AB, Canada; ^7^ Department of Chemistry and Biochemistry, University of Montana, Missoula, MT, United States; ^8^ Department of Environmental Science, Princeton University, Princeton, NJ, United States; ^9^ Department of Biochemistry and Molecular Biology, University of Southern Denmark, Odense, Denmark; ^10^ Clodomiro Picado Institute, University of Costa Rica, San José, Costa Rica

**Keywords:** cross-linking (XL), mass spectrometry, immunoglobulin fold, structural biology, toxin neutralisation, protein inhibitor, snake envenomation, antiophidic activity

## Abstract

DM64 is a toxin-neutralizing serum glycoprotein isolated from *Didelphis aurita*, an ophiophagous marsupial naturally resistant to snake envenomation. This 64 kDa antitoxin targets myotoxic phospholipases A_2_, which account for most local tissue damage of viperid snakebites. We investigated the noncovalent complex formed between native DM64 and myotoxin II, a myotoxic phospholipase-like protein from *Bothrops asper* venom. Analytical ultracentrifugation (AUC) and size exclusion chromatography indicated that DM64 is monomeric in solution and binds equimolar amounts of the toxin. Attempts to crystallize native DM64 for X-ray diffraction were unsuccessful. Obtaining recombinant protein to pursue structural studies was also challenging. Classical molecular modeling techniques were impaired by the lack of templates with more than 25% sequence identity with DM64. An integrative structural biology approach was then applied to generate a three-dimensional model of the inhibitor bound to myotoxin II. I-TASSER individually modeled the five immunoglobulin-like domains of DM64. Distance constraints generated by cross-linking mass spectrometry of the complex guided the docking of DM64 domains to the crystal structure of myotoxin II, using Rosetta. AUC, small-angle X-ray scattering (SAXS), molecular modeling, and molecular dynamics simulations indicated that the DM64-myotoxin II complex is structured, shows flexibility, and has an anisotropic shape. Inter-protein cross-links and limited hydrolysis analyses shed light on the inhibitor’s regions involved with toxin interaction, revealing the critical participation of the first, third, and fifth domains of DM64. Our data showed that the fifth domain of DM64 binds to myotoxin II amino-terminal and beta-wing regions. The third domain of the inhibitor acts in a complementary way to the fifth domain. Their binding to these toxin regions presumably precludes dimerization, thus interfering with toxicity, which is related to the quaternary structure of the toxin. The first domain of DM64 interacts with the functional site of the toxin putatively associated with membrane anchorage. We propose that both mechanisms concur to inhibit myotoxin II toxicity by DM64 binding. The present topological characterization of this toxin-antitoxin complex constitutes an essential step toward the rational design of novel peptide-based antivenom therapies targeting snake venom myotoxins.

## 1 Introduction

DM64 is made up of five immunoglobulin-like (Ig-like) domains and, to our knowledge, is the only mammalian inhibitor of snake venom myotoxic phospholipases A_2_ (PLA_2_) (Rocha et al., 2002; Rocha et al., 2017). This antitoxin is homologous to DM43, a three Ig-like domain-protein inhibitor grouped into the MEROPS I43 family of Ig-related proteins that binds snake venom metalloendopeptidases (SVMP) with nanomolar affinity (Neves-Ferreira et al., 2002; Rocha et al., 2009; Brand et al., 2012). Both toxin-scavenging proteins are homologous to human α1B-glycoprotein (Ishioka et al., 1986), a cysteine-rich secretory protein 3 (CRISP3) binding protein (Udby et al., 2004) that has been associated with several pathologic conditions (Clerc et al., 2016).

DM64 and DM43 constitute biochemical defenses employed by the South American marsupial *Didelphis aurita* to avoid the toxic activity of viperid snake venoms [reviewed in (Neves-Ferreira et al., 2009; Bastos et al., 2016; Neves-Ferreira et al., 2017)]. Since opossums are prey and predators of snakes, toxin resistance is a doubly advantageous evolutionary trait (Arbuckle et al., 2017). Despite the complex composition of viperid venoms (Gutiérrez et al., 2017), serum-based inhibitors seem restricted to PLA_2_ and SVMP toxin families, which suffices against the harmful effects of bothropic envenomation. Therefore, unveiling the molecular basis of such resistance might open important biotechnological perspectives.

The rational design of peptide-based antivenom therapeutics inspired by natural inhibitors of venom toxins is a long-sought goal that has been hampered by the lack of structural information on these proteins. The large immunoglobulin superfamily (CATH 2.60.40.10) is the most diverse regarding the number of structural domains (Bork et al., 1994; Sillitoe et al., 2019). The Ig fold is structurally very stable and shows remarkable plasticity in terms of binding specificity. Proteins that use this fold perform many functions related to immunological recognition, and they are also associated with several developmental and homeostatic phenomena (Bork et al., 1994; Natarajan et al., 2015). Among 7,053 unique PDB structures currently classified within this superfamily (CATH + release 4.3), none shares more than 25% sequence identity with DM64, precluding *in silico* structure prediction by homology modeling. The recently published AlphaFold 2 prediction algorithm (Jumper et al., 2021) may generate more accurate models of the Ig-like domains of DM64, but their correct spatial positioning remains challenging. Previous attempts to crystallize DM64 have failed, most likely due to the negative impact of its glycan moieties, preventing structural analysis by X-ray crystallography from being performed.

In such a challenging scenario, one can benefit from integrative structural strategies, where different lower-resolution techniques may be combined to generate complementary structural information on proteins or protein complexes. Mass spectrometry (MS) and associated methods, such as cross-linking (XL) and hydrogen-deuterium exchange (HDX), are among the most widely used integrative approaches. Such experimental results can be integrated into computational modeling/docking pipelines, substantially improving structural model accuracy (Liu and Heck, 2015; Faini et al., 2016; Koukos and Bonvin, 2019).

In this study, distance restraints from XL-MS provided unprecedented insights into the overall topology of the complex made by DM64 and myotoxin II from *Bothrops asper* venom. This basic toxin of ∼14 kDa is a catalytically-inactive PLA_2_ homolog (Lys49 variant, Group IIA) that induces significant local myotoxicity (Lomonte and Gutiérrez, 1989; Francis et al., 1991). Complementary information derived from limited proteolysis, analytical ultracentrifugation (AUC), small-angle X-ray scattering (SAXS), and molecular dynamics simulations improved our confidence in the proposed assembly model.

## 2 Materials and Methods

### 2.1 DM64 and Myotoxin II

Mature DM64 was isolated from *Didelphis aurita* serum as described (Rocha et al., 2002). For simplicity, the protein sequence without the signal peptide (residues 25–504 of Q8MIS3) was renumbered 1–480 in the present study. Dr. Paulo Sergio D’Andrea undertook wild animals’ capture and handling protocols (Laboratory of Biology and Parasitology of Wild Mammals Reservoirs, Oswaldo Cruz Institute), with the approval of the Federal Environment Agency (Permanent License 13373-1) and the Oswaldo Cruz Institute Ethics Commission on Animal Use (CEUA/IOC L-036/2018). All research activities carried out with the Brazilian genetic heritage were registered in the National System of Genetic Resource Management and Associated Traditional Knowledge (SisGen AF0A111). Myotoxin II (P24605) was isolated from *Bothrops asper* venom as published (Mora-Obando et al., 2014a). Protein quantitation was based on 280 nm molar extinction coefficients: DM64 57,005 M^−1^cm^−1^ and myotoxin II 21,275 M^−1^cm^−1^ (Pace et al., 1995).

### 2.2 Molecular Mass Determination by Mass Spectrometry

DM64 was desalted, concentrated, and introduced into the mass spectrometer by a system composed of a dual pump Agilent 1200 HPLC system, a Rheodyne manual injection valve, and a VALCO 10-port valve. Fifty picomoles of the inhibitor were initially applied to a Waters MassPREP micro desalting column (2.1 × 5.0 mm; 20 μm; 1,000 Å) previously equilibrated with 0.23% (v/v) formic acid in water. Sample application/desalting/concentration proceeded at a flow rate of 300 μL/min for 5 min using the aforementioned mobile phase. The column was then set in line with the second HPLC pump so that the sample could be eluted from the column directly into the mass spectrometer. In this case, the flow rate was set to 75 μL/min, and the eluents were 0.23% (v/v) formic acid in 95% water (mobile phase A) and 0.23% (v/v) formic acid in 95% acetonitrile (mobile phase B). Dimethyl sulfoxide (DMSO) at a level of 5% was added to both solutions to increase analyte charging (Iavarone and Williams, 2003). The column was equilibrated with 5% B for 1.5 min, followed by a linear gradient from 5 to 50% B lasting 13.5 min, and 50–95% B for 2.0 min. Sample elution was monitored online on a Waters Synapt G1 HDMS instrument set as follows: source voltages—capillary (3.5 kV), sampling cone (40.0 V), and extraction cone (4.0 V); temperatures—source (100 °C) and desolvation (250°C); MCP detector voltage (1,700 V). Data were submitted for analysis using the MassLynx software package (Waters). Background subtraction settings for “polynomial order”, “below curve (%)”, and “tolerance” were set to 25, 5, and 0.01, respectively. A representative “zoomed” 1,280–1,420 m*/z* range was then chosen for processing with MaxEnt 1, a maximum entropy deconvolution software (Ferrige et al., 1992). Regarding MaxEnt 1 settings, the resolution was set for 0.50 Da/channel for a “uniform Gaussian damage model” with minimum intensity ratios of 33% (left and right) and allowing the algorithm to iterate until convergence. DM64 had a final molecular mass range iteration across 64,000 to 67,000 with a width at half height of 1.00 Da.

### 2.3 Analytical Ultracentrifugation

Oligomerization properties of DM64 and complex formation of DM64 with myotoxin II were studied by sedimentation velocity. DM64 and myotoxin II control experiments were performed at three different loading concentrations to examine the potential for mass action-induced oligomerization. For DM64, the concentration range spanned roughly one order of magnitude: 0.81 µM (measured at 230 nm), 4.2, and 9.0 µM (measured at 280 nm). For myotoxin II, we examined 25.7 µM (measured at 280 nm), 82.6, and 119 µM (measured at 295 nm). Mixtures of both proteins were measured at three molar ratios (1:1, 2:1, and 4:1 myotoxin:DM64, all measured at 230 nm). Sedimentation experiments were performed on a Beckman Proteomelab XLI AUC at the Center for Analytical Ultracentrifugation of Macromolecular Assemblies (CAUMA) at the University of Texas Health Science Center at San Antonio (UTHSCSA), and on a Beckman Optima AUC instrument at the Canadian Center for Hydrodynamics at the University of Lethbridge. All samples were measured at 20°C and 55 krpm by UV intensity detection using an An60Ti rotor and standard 2-channel titanium centerpieces (Nanolytics, Potsdam, Germany) or standard 2-channel epon-filled centerpieces (Beckman-Coulter, Indianapolis). All data were analyzed with UltraScan 4.0 release 6,153 (Demeler and Gorbet, 2016). All samples were measured in a 10 mM sodium phosphate buffer containing 50 mM NaCl, pH 7.4. Hydrodynamic buffer density (1.0012 g/cm^3^) and viscosity (1.0065 cp) were estimated with UltraScan (Demeler and Gorbet, 2016).

Sedimentation velocity (SV) data were analyzed as reported earlier (Demeler, 2010). Optimization was performed by 2-dimensional spectrum analysis [2DSA, (Brookes et al., 2010)] with simultaneous removal of time- and radially invariant noise contributions and fitting of boundary conditions. 2DSA solutions were subjected to parsimonious regularization by genetic algorithm analysis (Brookes and Demeler, 2007) and, where applicable, by the enhanced van Holde–Weischet analysis (Demeler and van Holde, 2004). A further refinement using Monte Carlo analysis (Demeler and Brookes, 2008) was also applied to determine confidence limits for the determined parameters. The calculations were carried out on high-performance computing platforms at the Texas Advanced Computing Center and the San Diego Supercomputing Center (Brookes and Demeler, 2008).

For hydrodynamic radius (R_h_) estimates, SV experiments were fitted to finite element solutions of the Lamm equation to obtain a solute’s sedimentation and diffusion coefficients from the experimental data (Cao and Demeler, 2008). The diffusion coefficient, D, is given by:

$$D=\frac{RT}{Nf}$$

Where *R* is the universal gas constant, *T* is the absolute temperature, *N* is Avogadro’s number, and *f* is the translational frictional coefficient of the solute. Combining the diffusion coefficient with the Stokes-Einstein relationship (Einstein, 1905) allows us to calculate the hydrodynamic radius, *R*_*h*_:

$$R_{h}=\frac{RT}{6\pi \eta ND}$$

Where *η* is the viscosity of the solvent.

### 2.4 Size Exclusion Chromatography (SEC)

The stoichiometry of the interaction between DM64 and myotoxin II was analyzed by SEC, as described (Bastos et al., 2020), with modifications. A fixed amount of DM64 was titrated with increasing concentrations of myotoxin II (1:0.5; 1:1; 1:2; 1:4 mol/mol) in 0.1 M Tris-HCl pH 7.5, containing 0.5 M NaCl. The mixtures were incubated at 25°C for 15 min and then injected on a Superdex 200 Increase 5/150 GL column (GE Healthcare), previously equilibrated with the same buffer. The chromatogram peaks were integrated, and the areas corresponding to free myotoxin or to free DM64 co-eluted with DM64-myotoxin complex were plotted. The experiment was run in duplicate.

### 2.5 Small-Angle X-Ray Scattering

SAXS data were collected at the SAXS2 beamline (Brazilian Synchrotron Light Laboratory, Campinas, Brazil). The radiation wavelength was set to 1.48 Å. The scattering vector ranged from 0.1 to 1.5 nm^−1^. DM64 was mixed with myotoxin II (1:1 mol/mol) in 20 mM Tris-HCl pH 7.5, containing 20 mM CaCl_2_ and 150 mM NaCl. Frames with an exposure time of 300 or 600 s were recorded. To guarantee an accurate solvent correction, buffer baselines were collected under identical conditions before and after sample data collection. Background scattering was subtracted from the protein scattering pattern, which was then normalized and corrected. Data were processed and analyzed with the ATSAS package (Franke et al., 2017). Experimental data fitting and evaluation of the pair-distance distribution function P(r) were performed using the program GNOM (Svergun, 1992a). The radius of gyration (Rg) was estimated by the indirect Fourier transform method, and the maximum dimension Dmax was calculated from the actual space P(r) function as the distance r, where the P(r) value reaches zero. The theoretical scattering curve and radius of gyration of DM64-myotoxin complex were calculated using CRYSOL (Svergun et al., 1995). The comparison of Kratky plots for free DM64 and the complex DM64-myotoxin II was performed normalizing the scattering data for I (0) = 1 and multiplying q by Rg in order to remove the protein size information and keep the shape information (Kikhney and Svergun, 2015).

### 2.6 Cross-Linking Reaction

DM64 (20 µg) and myotoxin II were dissolved in 20 mM HEPES pH 7.5 and incubated at 25°C for 15 min (1:1 and 1:2 mol/mol). The noncovalent complex formed between both proteins was stabilized with BS^3^ (bis(sulfosuccinimidyl)suberate, Thermo Fisher Scientific) for 90 min at 25°C. The reaction was stopped by quenching in 20 mM ammonium bicarbonate. Protein (5–10 µM final concentration) to cross-linker ratios ranging from 1:500 to 1:2,800 mol/mol (the latter corresponding to 1:20 w/w) were tested. DM64 and myotoxin II controls were individually submitted to the same experimental procedure, using the higher protein to cross-linker ratio. Aliquots (10%) of each sample were analyzed by native polyacrylamide gel electrophoresis (PAGE) and sodium dodecyl sulfate (SDS-PAGE) under reducing conditions (12%T, silver staining) (Laemmli, 1970; Heukeshoven and Dernick, 1985). Molecular mass markers were from GE Healthcare. The remaining aliquots (90%) were injected on a Superdex 200 HR 10/30 column (GE Healthcare) to remove excess BS^3^ and protein aggregates. Finally, the samples were digested in solution with Lys-C endopeptidase (1:100 E:S, w/w) and trypsin (1:50 E:S, w/w) as previously described (Bastos et al., 2020). The experiment was run in duplicate.

### 2.7 Interaction Between Cross-linked DM64 and Native Myotoxin II

The functional integrity of DM64 cross-linked with BS^3^ was inferred based on its ability to interact with native myotoxin II, following incubation at 25°C for 15 min (1:1 mol/mol). Electrophoretic mobility shift assay on nondenaturing polyacrylamide gels was used to monitor complex formation. Protein bands were excised from gels, destained in 50% acetonitrile/25 mM ammonium bicarbonate pH 8.0, and further submitted to in-gel digestion (Shevchenko et al., 1996), modified as follows: 1) protein reduction was performed in 65 mM DTT in 100 mM NH_4_HCO_3_ pH 8.0 for 30 min at 56°C; 2) proteins were alkylated in 200 mM iodoacetamide in 100 mM NH_4_HCO_3_ pH 8.0 for 30 min at ambient temperature in the dark; 3) gel bands were swollen in a digestion buffer containing 40 mM NH_4_HCO_3_ pH 8.0 and 20 ng/μL trypsin (Promega V511); 4) for peptide extraction, gel bands were initially subjected to an ultrasonic bath treatment for 10 min, followed by vortexing for 20 s before transferring the supernatant volume to a new tube. An additional extraction step was performed following the addition of 30 µL of 5% formic acid in 50% acetonitrile to the gel bands, which were sequentially submitted to vortexing (for 20 s), incubation at ambient temperature (15 min), ultrasonic treatment (2 min) and vortexing (20 s). This extraction cycle was repeated one more time. Recovered supernatants were combined and dried on a vacuum centrifuge. Dried samples were redissolved in 1% formic acid before peptide identification by MS/MS on a QExactive Plus orbitrap (Thermo Scientific) (Fioramonte et al., 2018).

PatternLab for Proteomics V (PLV) (Carvalho et al., 2016) was used for peptide-spectrum matching and filtering, using a database consisting of the sequences of DM64 and myotoxin II included in the PLV standard contaminant library (total of 125 target sequences). Database searches considered semi-tryptic specificity, maximum of two missed cleavages, and the following modifications: fixed carbamidomethylation of cysteine (+57.02146 Da), variable oxidation of methionine (+15.9949 Da), and variable hydrolyzed BS^3^ (dead-end) in lysine, serine, and N-terminal (+156.0786 Da). The results were filtered for a maximum error of 10 ppm (precursor and fragment-ions), accepting an FDR of 1%.

### 2.8 Mass Spectrometry Analysis of Cross-Linked Peptides

Cross-linked peptides were analyzed by nLC-nESI-MS/MS on a Dionex UltiMate™ 3,000 RSLCnano system coupled online to a QExactive Plus orbitrap mass spectrometer (Thermo Scientific) (Fioramonte et al., 2018), modified as follows: 1) a PicoFrit column (75 µm inner diameter, 15 µm tip) (New Objective) packed in-house (40 cm length) with ReproSil-PurC18-AQ (1.9 μm resin, 200 Å pore size) (Dr. Maisch GmbH) was used in all chromatographic runs; 2) Higher-energy Collisional Dissociation (HCD) was performed with stepped Normalized Collision Energy (sNCE) at 30 and 40. At least two technical replicates of each biological replicate were analyzed. The software *Spectrum Identification Machine for Cross-linked Peptides* (SIM-XL) (Lima et al., 2015; Fioramonte et al., 2018) was used. It searched uninterpreted MS/MS raw files against a local database, which comprised the FASTA sequences of DM64 and mature myotoxin II, with no decoy sequences included. The following parameters were used: full enzymatic specificity (Lys-C + trypsin), ≤3 missed cleavages allowed, mass tolerance filters of 5 ppm for both MS1 and MS2, fixed modification of cysteine residues (carbamidomethyl, +57.02146 Da), and hydrolyzed BS^3^ dead-end (+156.0786 Da) as variable modification. A mass shift of +138.068100 Da was assumed to be associated with peptides cross-linked with BS^3^. The software considered only MS/MS spectra that contained at least one of the following XL reporter ions: *m/z* 222.149, *m/z* 239.1759, *m/z* 240.159, or *m/z* 305.2229. The assumed reaction site specificities for BS^3^ were KK, KS, SS, KN-TERM, SN-TERM. Identified spectra were submitted to automatic filtering (SIM-XL score ≥3; annotated fragment-ions/peptide chain ≥3), followed by careful manual verification (Iacobucci and Sinz, 2017). All curated cross-links met the following criteria: 1) High signal-to-noise ratio spectrum, where only minor peaks were unassigned; 2) Good sequence coverage for both cross-linked chains; 3) Unequivocal positioning of the cross-linking site; 4) Homogeneous mass error distribution for fragment-ions.

### 2.9 Limited Protein Hydrolysis and Affinity Purification of Interacting Peptides

Hitrap® NHS-activated affinity column (1 ml) containing immobilized myotoxin II was prepared as described (Rocha et al., 2002). It was used as bait to fish potential interacting peptides generated by enzymatic hydrolysis of DM64. Briefly, DM64 (200 µg) was dissolved in 0.4 M ammonium bicarbonate and 8 M urea, reduced with DTT and alkylated with iodoacetamide (Camacho et al., 2016). Using a ZebaTM Spin column (Thermo, 7 KMWCO), the sample was desalted, the buffer was exchanged to Tris-HCl 25 mM pH 8.5 containing 1 mM EDTA, and protein digestion with Lys-C endopeptidase (Promega) proceeded for 20 h at 37°C (1:100 E:S, w/w). The hydrolysate was chromatographed through the affinity column coupled with the toxin. Bound and unbound peptide fractions were desalted on self-packed Poros® 20 R2 (Applied Biosystems) tip columns, dried on a vacuum centrifuge, redissolved in 1% formic, and further analyzed by MS/MS (Camacho et al., 2016). Each fraction was analyzed in technical duplicate.

Alternatively, affinity fractions were directly injected onto a Vydac C18 column (2.1 mm × 15 cm). The collected peaks were dried, redissolved in 1% formic acid, and individually analyzed by MS/MS. Reversed-phase chromatography was performed at 0.2 ml/min, employing 0.1% TFA (solvent A) and 0.1% TFA in acetonitrile (mobile phase B) and the following elution gradient: 5% B for 5 min, 45%B at 55 min, 75% B at 60 min. A similar approach was used for fishing myotoxin II peptides generated by Glu-C endopeptidase (Promega), with the following modifications: after denaturation, reduction and alkylation, the toxin was desalted on a C18 spin column (Harvard Apparatus) equilibrated with 25 mM ammonium carbonate pH 7.8; the toxin was enzymatically digested with Glu-C for 20 h at 25°C at a 1:20 E:S ratio (w/w); the affinity column containing immobilized DM64 was prepared as described (Rocha et al., 2017).

Peaks X algorithm (Bioinformatics Solutions Inc.) was used to search MS/MS spectra against a database consisting of DM64 and myotoxin II sequences, embedded in a set of 20,533 human proteins retrieved from SwissProt, in addition to common contaminants (https://www.thegpm.org/crap). The following parameters were used for database searching: error tolerance of 10 ppm and 0.02 Da respectively for precursor- and fragment-ions; up to two missed cleavages allowed and fully-tryptic cleavage specified; carbamidomethylation of cysteines (+57.02146 Da) as fixed modification and no variable modification included. Identification results were filtered to accept ≤1% FDR at the spectral, peptide, and protein levels.

### 2.10 Molecular Modeling

The amino acid sequence of DM64 (Q8MIS3) was retrieved from the Uniprot database. The structures of the mature inhibitor or its isolated five Ig-like domains were initially obtained through homology modeling calculations with the automated server “Iterative Threading ASSEmbly Refinement” (I-TASSER) (Roy et al., 2010; Yang et al., 2015). Then, models were visually inspected, and images were rendered using Visual Molecular Dynamics version 1.9.3 (Humphrey et al., 1996).

Cross-linked residues identified by mass spectrometry were converted into distance restraints, assigning a Euclidean distance between Cβ atoms of each pair of reactive residues. Euclidean and topological distances were calculated applying TopoLink v1.18 (Ferrari et al., 2019). The software output classified all cross-linking results, correlating Euclidean and topological distances and defining distance limits for each cross-link. For the cross-linking agent BS^3^, the maximum extended Euclidean distances (D^Euclid^) was defined as 1) Lys-Lys, 21.8 Å; 2) Lys-Ser, 18.0 Å; Ser-Ser, 14.1 Å.

### 2.11 Molecular Docking

The interaction between DM64 and myotoxin II was studied by molecular docking, applying “Rosetta Docking Protocol” (Gray et al., 2003; Wang et al., 2005) guided by distance constraints derived from cross-linking experiments. First, the crystal structure of the myotoxin II monomer was retrieved from the PDB databank under the identification code 1CLP (chain A). Next, myotoxin II structure and DM64 Ig-like domains’ models were relaxed with standard protocols by “Rosetta’s short Relax Protocol,” constraining backbone and side-chain heavy atoms based on the input structure and turning off-ramp down constrains. One hundred structures were generated for each protein, selecting the lowest total energy structure as input to the molecular docking (Nivon et al., 2013; Conway et al., 2014).

The complete view of the interaction between DM64 and myotoxin II was obtained in five steps: 1) docking D5-myoII, 2) docking D4-D5myoII, 3) docking D3-D4D5myoII, 4) docking D2-D3D4D5myoII, and 5) docking D1-D2D3D4D5myoII. Each docking step consisted of three phases: in the first one, a single PDB file was created manually, adding a pair of proteins (or a protein and a partial complex) displaced 20 Å away from each other; in the second phase, 150,000 runs of global docking were performed, aiming to generate an initial model for the interaction based on cross-linking distance restraints. In this phase, docking partners’ positions were spun and randomized before each run, avoiding a bias created by choosing initial coordinates for the docking. Docking models were analyzed by the TopoLink software and further classified by the number of violated constraints. The group with the lowest number of violated constraints was clustered according to each constraint (validated or not) sorted by Rosetta’s total energy. The lowest total energy score model was then chosen as a starting coordinate for the local docking step.

In the third phase, 150,000 runs of local docking were performed, sampling the conformational space around the previously selected conformation, searching for the best agreement with experimental data. In this phase, the first docking partner (myotoxin II alone or in the complex) was fixed, and the incoming DM64 domain was perturbed by 3 Å translation and 8° rotation before each run. TopoLink was then used to analyze models and classify them according to the number of violated constraints. The group with the lowest number of violated constraints was clustered according to each constraint (validated or not) and sorted by the Rosetta energy terms: TotalScore, InterfaceScore (I_sc), and ConstraintScore (AtomPair). The lowest ConstraintScore was chosen as the final docking model and used as starting coordinate for docking the next DM64 domain.

### 2.12 Molecular Dynamics Simulation

The docked model of the DM64-myotoxin II complex was used as starting coordinates for the molecular dynamics (MD) simulations. Simulations were performed by Gromacs version 2020.3 (Berendsen et al., 1995) with Amber99SB force field (Jorgensen et al., 1996) and TIP3P water model (Jorgensen et al., 1983). The Propka module determined the ionization states of side chains for DM64 and myotoxin II in the PDB2PQR server at pH 7.5 (Dolinsky et al., 2007). The starting model was centered in a cubic water box at 2 nm of the box limits in any dimension. The charges were neutralized with counterions.

Energy minimization was applied using steepest descent and conjugated gradient algorithms with a final maximum force of 1,000 kJ/mol/nm (0.01 nm step size, cutoff of 1.2 nm for neighbor list, Coulomb interactions, and van der Waals interactions). Before the production runs, sequential equilibration processes in NVT (1.0 ns) and NPT (1.0 ns) were performed to adjust the systems into the desired temperatures and volumes, under position restraints with the force constants of 1,000 kJ/(mol·nm^2^) applied to the protein complex. LINCS algorithm and virtual sites were used with a time step of 2 fs (cutoff of 1.4 nm neighbor list, Coulomb interactions, and van der Waals interactions). The temperature was stabilized by a V-rescale thermostat (Bussi et al., 2007), and pressure was coupled to a Parrinello-Rahman barostat (Parrinello and Rahman, 1981).

The position restraints were released gradually in 26 NVT equilibrium steps (200 ps each) with the force constants 1,000, 800, 600, 400, 300, 250, 200, 175, 150, 125, 100, 75, 50, 25, 23, 20, 18, 16, 14, 12, 10, 8, 6, 4, 2 and 0 kJ/(mol·nm2). After the preparation phase, 600 ns of MD simulation was accumulated and fitted, considering myotoxin II as the system’s center.

Gromacs native package was used to analyze: 1) root mean square deviation (RMSD); 2) root mean square fluctuation (RMSF) of atomic positions in the trajectory; RMSD cluster analysis using Gromos clustering algorithm with 2 nm cutoff; 4) radius of gyration (Rg), and 5) hydrogen bonds with a maximum distance of 3.5 Å between donor and acceptor and a maximum angle of 30° between donor, donor-hydrogen and acceptor. The percentual time a hydrogen bond was present in the simulation was extracted from the interprotein hydrogen bond existence matrix, using a bash script that extracts the total number of occurrences of a hydrogen bond in the total number of frames of the MD simulation.

Principal component analysis (PCA) evaluated the conformational space sampled in the MD simulation of the DM64-myotoxin II complex. First, Gromacs module “covar” was applied to calculate and diagonalize the (mass-weighted) covariance matrix. Second, Gromacs module “anaeig” was used to analyze the eigenvectors of the covariance matrix. The main conformational motions were represented by the projections of the first and second principal components (PC1 and PC2, respectively). Trajectories were visualized, and images were rendered using Visual Molecular Dynamics software version 1.9.3

## 3 Results

### 3.1 DM64-Myotoxin Interaction: Molecular Mass and Stoichiometry Analyses

**FIGURE 1:**
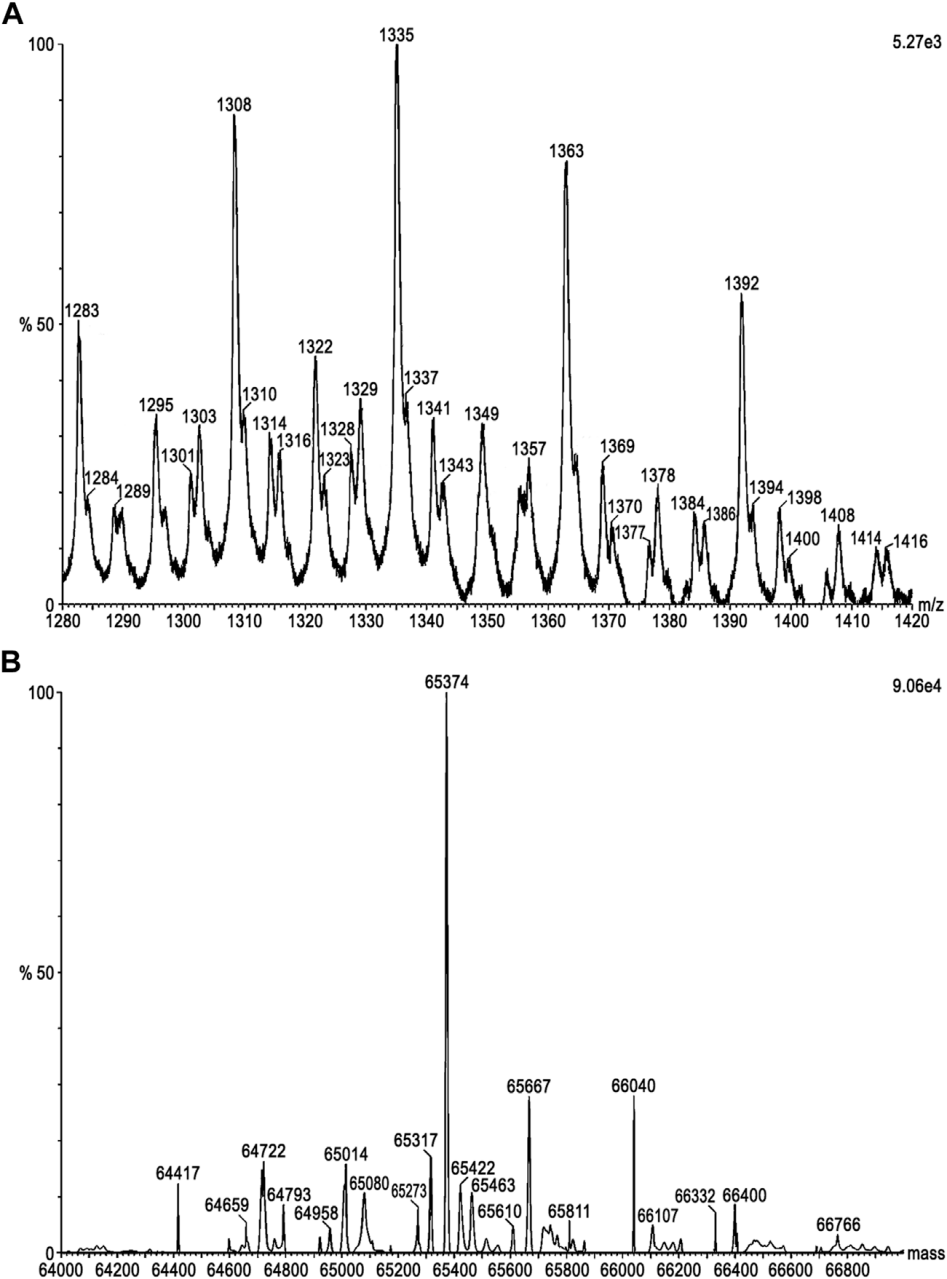
Molecular mass determination of DM64 by ESI-MS. The setup was composed of an Agilent 1200 HPLC system coupled to a Waters Synapt G1 mass spectrometer. DM64 (50 pmol) was desalted on a Waters MassPREP microdesalting column (2.1 × 5.0 mm; 20 μm; 1,000 Å) followed by direct elution into the ion source for MS analysis. Acquired data were submitted to analysis with the MassLynx software, including **(A)** background subtraction, choice of *m/z* range for further processing, and **(B)** deconvolution using MaxEnt 1 algorithm to generate the final mass values.

**FIGURE 2:**
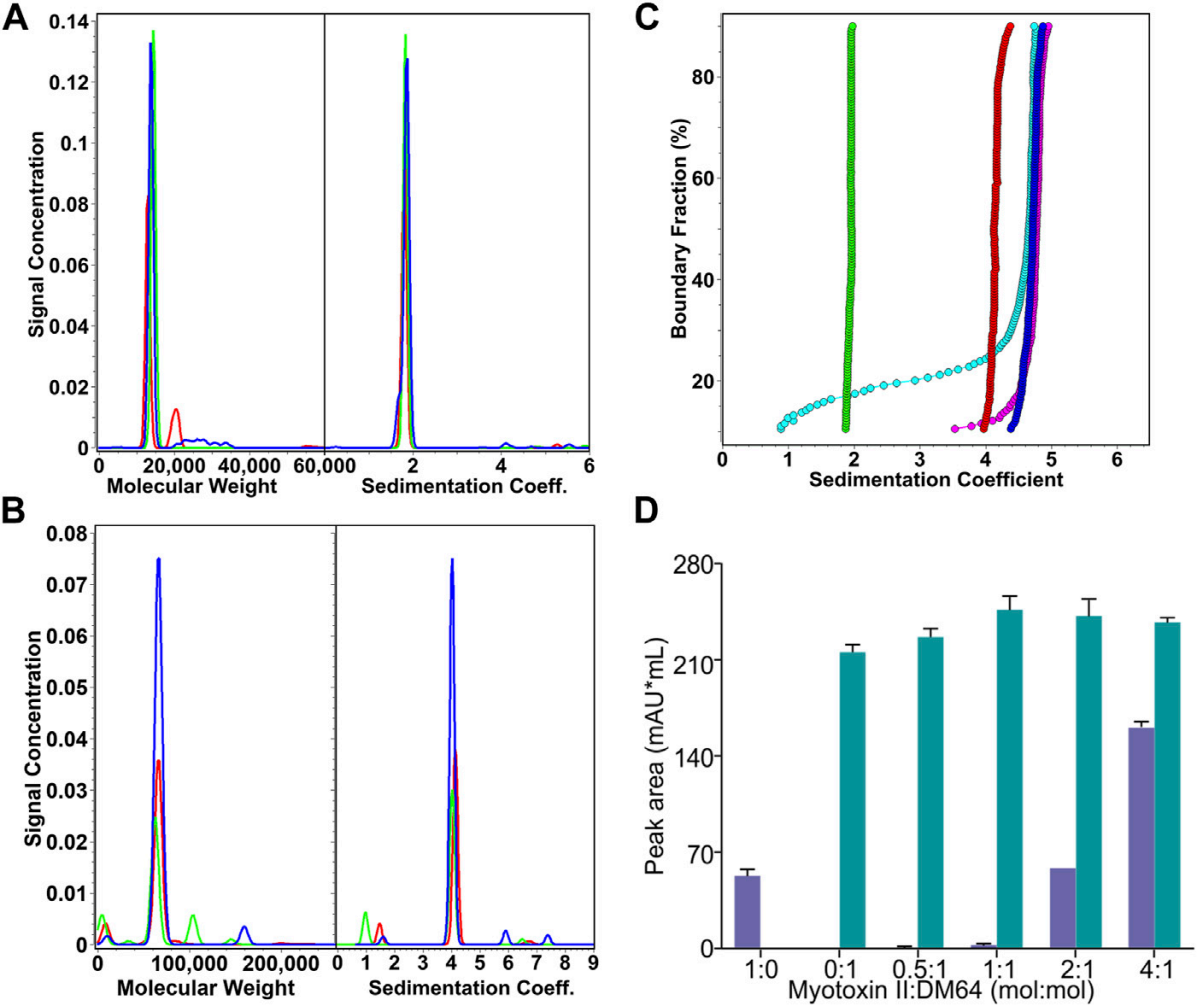
Analysis of the stoichiometry of the interaction between DM64 and myotoxin II. **(A)** Sedimentation coefficient distributions and molar mass estimates of myotoxin II controls (25.7 µM, green; 82.6 µM, red; 119 μM, blue). **(B)** Sedimentation coefficient distributions and molar mass estimates of DM64 controls (0.81 μM, red; 4.2 µM, green; 9.0 µM, blue) **(C)** van Holde-Weischet integral G(s) distributions of myotoxin II:DM64 mixtures (1:1, blue; 2:1, magenta; 4:1, cyan) and myotoxin II (green) and DM64 (red) monomeric controls. **(D)** Size exclusion chromatography analysis of myotoxin II and DM64 mixed at different molar ratios. The peak areas in the chromatograms were integrated and plotted. Because the retention times for DM64 alone and the complex DM64-myotoxin II are very similar, they were plotted as the sum of co-eluted peaks (green bars). Free myotoxin II eluted at a different elution time (blue bars).

The molecular mass of DM64 determined by ESI-Q-TOF was 65.37 kDa (Figures 1A,B). Sedimentation velocity experiments were performed to analyze the oligomerization state of DM64 and myotoxin II. AUC measurements resulted in molar masses in excellent agreement with the known molar masses of monomeric toxin and inhibitor (Table 1). Sedimentation profiles from increasing concentrations of those two proteins (Figures 2A,B) did not produce any change in the sedimentation behavior or molar mass, suggesting the absence of mass action in the examined concentration range.

**TABLE 1:** Molecular mass determination by sedimentation velocity experiments.

	Sedimentation coefficient (s, x10^−13^)	Diffusion coefficient (cm^2^/sec, x10^−7^)	Hydrodynamic radius (nm)	Molar mass (Dalton, x10^3^)	PSV (ml/g)	f/f_0_
Myotoxin II (60 µM)	1.84	12.10	1.77	13.5	0.7266	1.13
DM64 (1 µM)	4.03	5.41	3.96	63.0	0.7134	1.52
DM64:myotoxin II 1:1	4.84	5.08	4.22	81.5	0.7161	1.48

For DM64, glycosylation (∼18.5%) was expected to affect the partial specific volume. Using the ESI-Q-TOF result for DM64 (65.37 g/mol), the ExPASy computed average molar mass from protein sequence alone (53.3 g/mol, https://web.expasy.org/compute_pi), and the partial specific volume predicted with UltraScan based on protein sequence (0.7342 ccm/g for DM64 and 0.7266 ccm/g for myotoxin II), together with an average partial volume of 0.622 ccm/g for glycosylation reported by (Lewis and Junghans, 2000), we arrived at a modified partial specific volume of 0.7134 ml/g for the inhibitor, and 0.7161 ml/g for the 1:1 DM64-myotoxin II complex. Molar mass transformations based on the adjusted partial specific volumes are shown in Table 1.

When mixed at stoichiometric amounts, myotoxin II and DM64 formed a homogeneous species consistent with a 1:1 complex, and sedimented at 4.84 s, faster than the monomeric DM64 (4.03 s) and myotoxin II (1.84 s) controls. The 1:1 DM64:myotoxin II mixture was measured at a protein absorbance of 0.29 OD at 230 nm, corresponding to a molar concentration of 800 nM for each protein. This concentration is near the detection limit of the analytical ultracentrifuge as buffer interference increases at lower wavelengths. Our analysis clearly shows that all protein molecules are in a complex at this concentration (Figure 2C, dark blue line). A slower species representing either free DM64 or free myotoxin II could not be detected. When the relative molar ratio of the toxin was increased to 2:1 or 4:1, the same complex was observed with an unchanged sedimentation coefficient. Still, additional slower species, representing free myotoxin II, were observed (Figure 2C, cyan and magenta lines). These results indicate that the K_D_ is at most 800 nM, but most likely lower, reflecting a very strong interaction. DM64 binds with an equimolar amount of myotoxin II, and excess myotoxin II accumulates as free monomer. The molar mass transformation based on the adjusted partial specific volume for the 1:1 myotoxin II-DM64 complex is shown in Table 1. It is in excellent agreement with the sum of monomeric DM64 and monomeric myotoxin II.

The equimolar stoichiometry was corroborated by analytical size exclusion chromatography (Figure 2D). The peak area corresponding to co-eluting free DM64 and the toxin-antitoxin complex increased until a 1:1 myotoxin:DM64 molar ratio was reached. At a 2:1 myotoxin:DM64 molar ratio, no further increase in the area of the co-eluting peaks was detected; instead, a peak corresponding to free myotoxin was observed, and its area was equivalent to *ca.* 1-fold molar excess of myotoxin. When a 4:1 myotoxin-DM64 molar ratio was assayed, the peak area of the free toxin was proportional to *ca.* 3-fold molar excess of myotoxin II.

### 3.2 Mapping DM64-Myotoxin II Complex by Cross-Linking Mass Spectrometry

The complex made of DM64 and myotoxin II was stabilized using BS^3^. This cross-linker reacts with nucleophilic groups, such as primary amines and, to a lesser extent, hydroxyl groups (Sinz, 2006). Control samples of myotoxin II and DM64 were individually submitted to the same protocol. Using BS^3^, the number of cross-linkable residues (Lys + Ser) in DM64 represented 12% of the total number of residues. In contrast, in the very basic myotoxin II, the number of potential target residues was higher, covering 19.8% of the protein sequence.

To ensure high cross-linking yields with acceptable structural distortion, DM64 was initially reacted with increasing BS^3^ concentrations (1,000- to 2,800-fold molar excess over protein). Inference on protein fold integrity was based on the ability of cross-linked DM64 to interact with unmodified myotoxin II. Such interaction was monitored by nondenaturing gel electrophoresis (Supplementary Figure S1). Gel bands suspected to represent the toxin-antitoxin complex were analyzed by MS/MS. Under all experimental conditions, sequences corresponding to myotoxin II and DM64 were obtained from the same gel band. Thus, the comigration of toxin and antitoxin was interpreted as evidence of protein interaction. Such evidence suggests that eventual structural disturbances due to the chemical cross-linking of DM64 were somewhat limited.

To further optimize the experimental conditions, the toxin-antitoxin complex was stabilized with different concentrations of BS^3^. No significant artifactual aggregation/oligomerization was observed by native PAGE as the cross-linker concentration increased (Supplementary Figure S2A). On the other hand, the SDS-PAGE profile showed that the band broadening effect, typical of the cross-linking reaction (Leitner et al., 2014), was more pronounced when higher concentrations of BS^3^ were used (Supplementary Figure S2B). Therefore, all subsequent cross-linking experiments were performed with the highest molar excess of this cross-linker.

**FIGURE 3:**
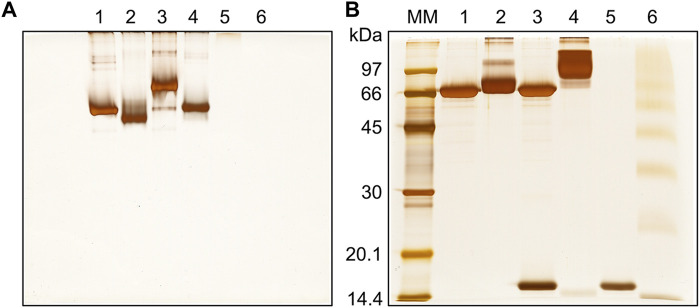
Gel electrophoresis analysis of the cross-linking reaction between DM64 and myotoxin II (1:1 mol/mol). The toxin-antitoxin noncovalent complex was stabilized with BS^3^ (90 min reaction time at 25°C, protein to cross-linker ratio of 1:2,800 mol/mol) and analyzed by **(A)** native PAGE and **(B)** SDS-PAGE under reducing conditions. All samples were run on 12% T gels stained with silver nitrate. MM: molecular mass markers; lane 1: DM64; lane 2: DM64 + BS^3^; lane 3: DM64 + myotoxin II (mtx II); lane 4: (DM64 + mtx II) + BS^3^; lane 5: mtx II; lane 6: mtx II + BS^3^.

BS^3^-stabilized toxin-antitoxin complex migrated as a single sharp band on the native gel, with slower mobility than cross-linked DM64 (Figure 3A). Due to its basic nature (pI > 9) (Lomonte and Rangel, 2012), control myotoxin II did not enter the stacking gel (Figure 3A, lanes 5–6). The complex stabilized with BS^3^ was visualized as a broad higher molecular mass band on reducing SDS-PAGE, and virtually no bands corresponding to free myotoxin or DM64 could be observed (Figure 3B, lane 4). The high number of lysines in myotoxin II led to several higher-order oligomers when the toxin was cross-linked with BS^3^ in the absence of DM64 (Figure 3B, lane 6), thus precluding the subsequent analyses of this sample.

**FIGURE 4:**
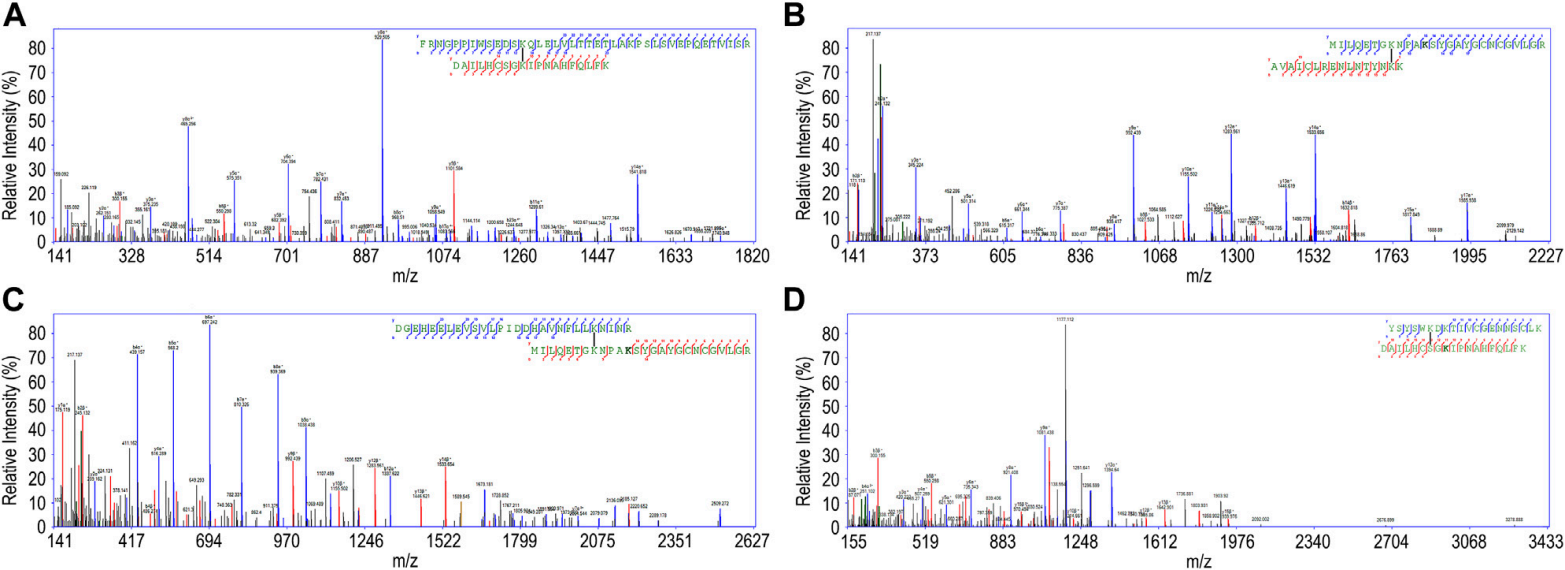
Representative good-quality fragmentation spectra of peptides cross-linked with BS^3^, interpreted by the SIM-XL software, and manually verified. **(A)** Intra-protein link from DM64 (scan # 27,856, SIM-XL primary score 6.74); **(B)** Intra-protein link from myotoxin II (scan # 20,073, score 4.62); Inter-protein link from DM64-myotoxin II complex: **(C)** Scan # 28,261, score 5.86; **(D)** Scan # 21,754, score 4.32. For each spectrum, the sequences of the cross-linked peptides are shown, along with all observed b- and y-ions (blue and red lines, respectively for α- and β-peptides) and the position of the linker (black line).

Several spectra corresponding to cross-linked peptides from the complex made of DM64-and myotoxin II were identified by the SIM-XL software (representative examples in Figure 4). They were manually inspected to allow the selection of high-confidence cross-linking-spectrum matches (CSM). Sequence coverages of the DM64-myotoxin II complex, based on the primary structures of intra- (type 1) and inter- (type 2) cross-linked peptides (Schilling et al., 2003), were: 84.3% for myotoxin II and 69.8% for DM64 (not shown). In the case of DM64, non-covered regions corresponded to the surrounding sequences of the four N-glycosylation sites. Similar results were obtained when DM64 was cross-linked without the toxin.

Ideally, cross-links should be validated on experimentally determined high-resolution structures before targeting unknown 3D structures (Merkley et al., 2014). Therefore, as a proof-of-principle test, all manually curated intra-myotoxin II BS^3^ links (observed when the toxin was in complex with DM64) were mapped on the crystal structure of the toxin (1CLP, chain A) (Supplementary Figure S3). No aberrant cross-links could be detected, although 4 out of 15 unique links (73% validation) exceeded the maximum allowed topological distance between the β-carbon of cross-linked residues, calculated from the X-ray structure (Supplementary Tables S1A,C). They involve residues located in regions of the toxin with higher average B-factor values and may reflect the structural flexibility of the protein in solution.

**FIGURE 5:**
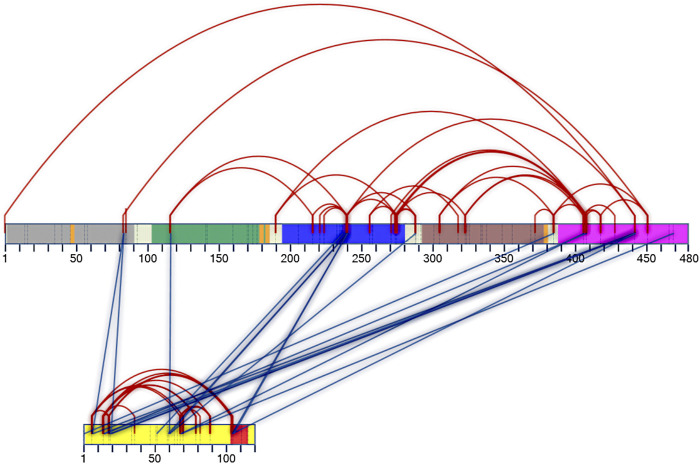
Two-dimensional interaction map of DM64 in complex with myotoxin II. Protein sequences are represented by numbered rectangles in which vertical dashed lines indicate all potential target residues for cross-linking reaction. DM64 protein is colored according to Ig-like domains: D1: gray; D2: green; D3: blue; D4: brown; D5: pink. The four consensus sequences for N-glycosylation are shown in orange, and inter-domain linker regions are shown in white. The protein sequence of myotoxin II is represented by a yellow rectangle, with the C-terminal cationic/hydrophobic myotoxic site colored in red. Intra-protein cross-links are represented by red lines, whereas inter-protein cross-links are shown in blue. Following identification by the SIM-XL software and manual verification, only good-quality spectra were used to build the 2D map.

Regarding the connections within DM64, 32 unique cross-links were identified when the protein was complexed with myotoxin II (red lines in Figure 5 and Supplementary Table S1D). A similar pattern was observed when DM64 was cross-linked in its free form (not shown), and 39 unique cross-links were confidently identified (Supplementary Table S1E). In both cases, several unique cross-links connected residues far apart in the primary structure of the inhibitor, clearly indicating the close spatial proximity of the corresponding cross-linking sites.

Eighteen unique inter-protein cross-links were confidently identified between DM64 and myotoxin II (blue lines in Figure 5 and Supplementary Table S1F). Half of the links connected the fifth domain of DM64 (residues Lys386, Ser407, Lys409, Lys443, and Ser470) with myotoxin II, five of which were centered at Lys443, a cross-linking hotspot in the inhibitor sequence. Most cross-linked residues in the fifth domain of DM64 (6 out of 9 cross-links) connected the inhibitor to the N-terminal region of the toxin. The third domain of DM64 showed the second-highest number of cross-links with myotoxin II. In total, five cross-links were confidently identified, almost always involving the inhibitor’s residue Lys241 and the middle (Lys60 and Lys61) or the C-terminal (Lys105 and Lys106) regions of the toxin.

### 3.3 Identification of Potential Interaction Regions Between DM64 and Myotoxin II

The myotoxin II hydrolysate bound to the DM64 affinity column was subjected to off-line C18 reversed-phase fractionation, and the collected fractions were identified by MS/MS. Only one toxin peptide, corresponding to the C-terminal end (99-NLNTYNKKYRYYLKPLCKKADAC-121), was confidently identified. The same result was obtained when the bound fraction was directly analyzed by MS/MS, without pre-fractionation. Similarly, using both methodological strategies, one DM64 peptide was identified in the hydrolysate bound to myotoxin II. Its sequence was located in the fifth domain of the inhibitor (420-DGEHEELEVSVLPIDDHAVNFLLK-443). Two other DM64 peptides were confidently identified only when MS/MS directly analyzed the bound hydrolysate: one peptide located in the third domain of the inhibitor (258-YSCRYRFRNGPPIWSEDSK-276) and a second one corresponding to its C-terminal end (453-YRCRYTTREDPILESEMSDPAELQVTGQ-480). All peptides bound to the affinity columns were deemed too long to be used as constraints in the docking strategies. Nevertheless, they served as additional evidence to further validate the toxin-antitoxin model, as discussed later.

### 3.4 Molecular Modeling

#### 3.4.1 DM64: Complete Primary Structure

In a first attempt, DM64 was modeled based on its whole amino acid sequence, applying the algorithms I-TASSER (Yang et al., 2015) or Rosetta (Yang et al., 2020). The models produced by each strategy were convergent, showing an extended spatial distribution of the five Ig-like domains (not shown). All models were then submitted to topological validation using experimentally derived structural data on the isolated inhibitor generated by cross-linking mass spectrometry. The model with the highest number of validated cross-links was chosen to represent this strategy.

Intra-domain cross-links (13 of 39 observed cross-links) were used to validate individual Ig folds (Supplementary Table S1B). In contrast, inter-domain cross-links (26 out of 39) were used to analyze the model’s global structure. In the first case, an overall validation rate of 61.5% was obtained, as follows: 75.0% validation within the third domain of DM64 (3 validated links/4 observed links); 0% in D4 (0 validation/1 observation); 62.5% in D5 (5 validations/8 observations). Regarding inter-domain cross-links, only one could be validated out of 26 identified links (3.8%).

#### 3.4.2 DM64: Individual Ig-like Domains

The modeling strategy was further optimized to incorporate myotoxin II structure as a reference point to guide the spatial positioning of DM64 domains. Initially, individual DM64 Ig-like domains were modeled applying the same algorithms used for the whole protein. Each domain extension was defined in this first step based on the Ig-like fold, and the transition regions for consecutive domains were identified. Supplementary Figure S4 shows the primary structure of DM64, with residues delimiting the N- and C-terminal regions of each domain colored in yellow. Conserved connecting regions that allow flexibility and plasticity between the domains could be identified for each domain pair and are highlighted in cyan. The sequence of each Ig-like domain was then adjusted to include four to five residues of the connecting region, including a security range in each domain’s terminal region. Table 2 shows the final extension of all modeled domains, together with their main structural features. Next, individually modeled domains were used to build the model structure of DM64 bound to myotoxin II, following a series of molecular docking steps guided by distance restraints generated by cross-linking mass spectrometry. Only cross-links observed in the toxin-complexed inhibitor were considered.

**TABLE 2:** Extension and main characteristics of the immunoglobulin-like domains of DM64.

Domain	Start	End	Cys-Cys	Glycosylation site	Residues
D1	001-LAMET-005	091-VTGKE-095	027–073	019-PWT**N**VTL-025	95
D2	099-APLLR-103	183-VVIPD-187	120–162	155-**N**NTG**N**YS-161	89
D3	191-KPDFH-195	281-VLTTE-285	212–260	—	95
D4	289-KPSLS-293	377-EIRVE-381	311–358	351-YDTG**N**FS-357	93
D5	386-KPTLH-390	476-QVTGQ-480	406–455	—	95

Domain, Sequential name of DM64 domains; Start, starting sequence and residue number for each domain; End, ending sequence and residue number for each domain; Cys-Cys, disulfide bond identified by residue numbers; Glycosylation Site, N-glycosylation sites in consensus sequence (**N**X-S/T) and residue numbering; Residues, number of residues in the domain.

### 3.5 Molecular Docking Between Individual Ig-like Domains of DM64 and Myotoxin II

For the sake of clarity during the molecular docking process, each individual Ig-like domain of DM64 was indexed by a letter, as follows: D1 (chain A), D2 (chain B), D3 (chain C), D4 (chain D), D5 (chain E). Myotoxin II (myoII) was referred to as chain F. To facilitate interpreting the results, all main structural features of the myotoxin II crystal structure were highlighted in the ribbon representation shown in Supplementary Figure S5. Detailed results of all docking steps performed between DM64 and myotoxin II described below are shown in Supplementary Table S1A.

#### 3.5.1 First Docking Step: D5-Myotoxin II

Nine cross-links were identified between the fifth domain of DM64 and myotoxin II (Supplementary Figure S6), the second-highest number of cross-links observed. Only two were not validated in the model structure generated by molecular docking: Lys386E-Ser1F (N-terminal residue of myotoxin II) and Lys409E-Lys15F (residue located in the small helix of myotoxin II). On the other hand, the remaining seven observed cross-links were topologically validated using the same reference structure, yielding an overall validation rate of 77.8%. The docking results indicate that D5 is spatially close to the N-terminal helix of myotoxin II (cross-link observed between Lys443E-Lys7F) and the small helix region (Lys443E-Lys15F, Lys443E-Lys19F, and Lys443E-Ser20F). D5 is likely also close to the beta-wing region (Ser407E-Lys69F and Ser470E-Lys69F) (Arni and Ward, 1995). Interestingly, the topologically validated cross-link Lys443E-Lys105F approximates D5 and myotoxin Lys105, the first residue of the myotoxic peptide located in the C-terminal region of the toxin.

#### 3.5.2 Second Docking Step: D4-D5Myotoxin II

Of six cross-links involving the fourth domain of DM64, four could be topologically validated in the available model structure (66.7% validation rate) (Supplementary Figure S7). A single cross-link was identified between D4 and myotoxin II: Lys289D-Lys61F was spaced by a valid topological distance connecting D4 and the frontal loop of the toxin. This region leads to the beta-wing, which is implicated in the interaction between myotoxin II monomers. The remaining five cross-links connect the fourth and the fifth domains of DM64, three of which were topologically validated (Lys306D-Lys386E, Lys324D-Ser407E, and Lys324D-Lys409E). It is worth mentioning that, in the final model of the complexed toxin-antitoxin structure, the N-glycosylation site in the fourth domain of DM64 (Asn355) (Table 2) was exposed at the surface as expected.

The docking models did not include the transition sequence between the fourth and fifth domains of DM64 (residues 382-GLLP-385) (Supplementary Figure S4, underlined residues). Nevertheless, when considering an entirely extended conformation, the expected maximum distance between Cα atoms of residues Glu381 and Lys386 in DM64 is 15.87 Å. This distance corresponded to 14.33 Å in the final docked model, thus compatible with the proposed covalent topology of the molecule.

#### 3.5.3 Third Docking Step: D3-D4D5Myotoxin II

The third domain of DM64 showed the highest density of unique cross-links. Nine out of 15 distance restraints were topologically validated, corresponding to an overall validity rate of 60% (Supplementary Figure S8). Five inter-protein cross-links connected D3 and two distinct regions of myotoxin II. Three cross-links (Lys238C-Lys52F, Lys241C-Lys60F, and Lys241C-Lys61F) comprise the toxin’s frontal loop and could be topologically validated. On the other hand, both cross-links (Lys241C-Lys105F and Lys241C-Lys106F) connecting the inhibitor and the first and second residues of the myotoxic C-terminal peptide did not fall below the distance limit imposed by the BS^3^ cross-linker.

Ten out of the 15 cross-links observed in D3 were classified as intra-protein. Five of them connected D3 to D4, including three validated links (Lys241C-Lys289D, Ser275C-Lys289D, and Lys276C-Lys289D) and two non-validated ones (Lys257C-Lys319D and Lys276C-Lys324D). The remaining five intra-protein cross-links were observed between D3 and D5. Three (Lys191C-Lys409E, Ser275C-Lys409E, and Lys276C-Lys409E) were within an acceptable topological distance, while two cross-links (Lys241C-Lys443E and Ser272C-Lys409E) did not comply with the model structure of the inhibitor. For all violated distances within DM64, measured Euclidean distances between the beta-carbons of cross-linked residues were under 34 Å.

The modeled structures of the inhibitor did not include the sequence between D3 and D4 (residues 286-TLA-288) (Supplementary Figure S4, underlined residues). The Cα-Cα distance between Glu285 and Lys289 in the fully extended conformation is 14.5 Å. This measurement in the docking model corresponded to 19.96 Å, thus lightly exceeding the maximum expected distance.

#### 3.5.4 Fourth Docking Step: D2-D3D4D5Myotoxin II

D2 encloses two glycosylation sites (Table 2) and showed the lowest number of experimental restrictions. Only three unique cross-links were identified, all of which centered at Lys117 and in good agreement with the maximum bound distances defined by the docked structure of the toxin-antitoxin complex (Supplementary Figure S9). A single inter-protein restriction (Lys117B-Lys61F) connected D2 to the frontal loop of myotoxin II. Two other cross-links within DM64 linked D2 to D3 (Lys117B-Ser217C and Lys117B-Lys241C). As expected, considering the presence of two N-linked glycan antennas, both glycosylation sites in the second domain of DM64 (Asn155 and Asn159) were exposed on the complex surface.

The transition sequence between D2 and D3 (residues 188-LLP-190) was not included in the docking models. In the fully extended conformation, the maximum distance between the alpha-carbons of Asp187 and Lys191 was 12.41 Å, whereas, in the final docking model, the two residues were 15.18 Å apart. Despite exceeding the distance limit, both N- and C- terminal regions of D2 and D3 are not structured, allowing certain flexibility regarding the maximum distance limit.

#### 3.5.5 Fifth Docking Step: D1-D2D3D4D5Myotoxin II

Four unique cross-links were identified in the N-terminal domain of DM64, three of which could be validated in the final docking model (75% validation) (Supplementary Figure S10). Two valid inter-protein cross-links (Ser84A-Lys7F and Ser84A-Ser20F) connected D1 and the N-terminal region of myotoxin II (residues Lys7 and Ser20, located at the N-terminal helix and the small helix, respectively). Two cross-links were identified within DM64, connecting D1 and D5: the topologically validated Leu1A-Lys443E and the non-validated Ser84A-Lys452E. Finally, the glycosylation site Asn22 (Table 2) was exposed at the complex surface in the final docking model, allowing the attachment of a glycan antenna.

The transition region between D1 and D2 (96-PLP-98) was not present in the docking models. Although a maximum distance of 10.67 Å was expected between the alpha-carbons of Glu95 and Ala99, the final docking model showed a larger maximum distance of 23.03 Å.

### 3.6 DM64-Myotoxin II Model Construction

**FIGURE 6:**
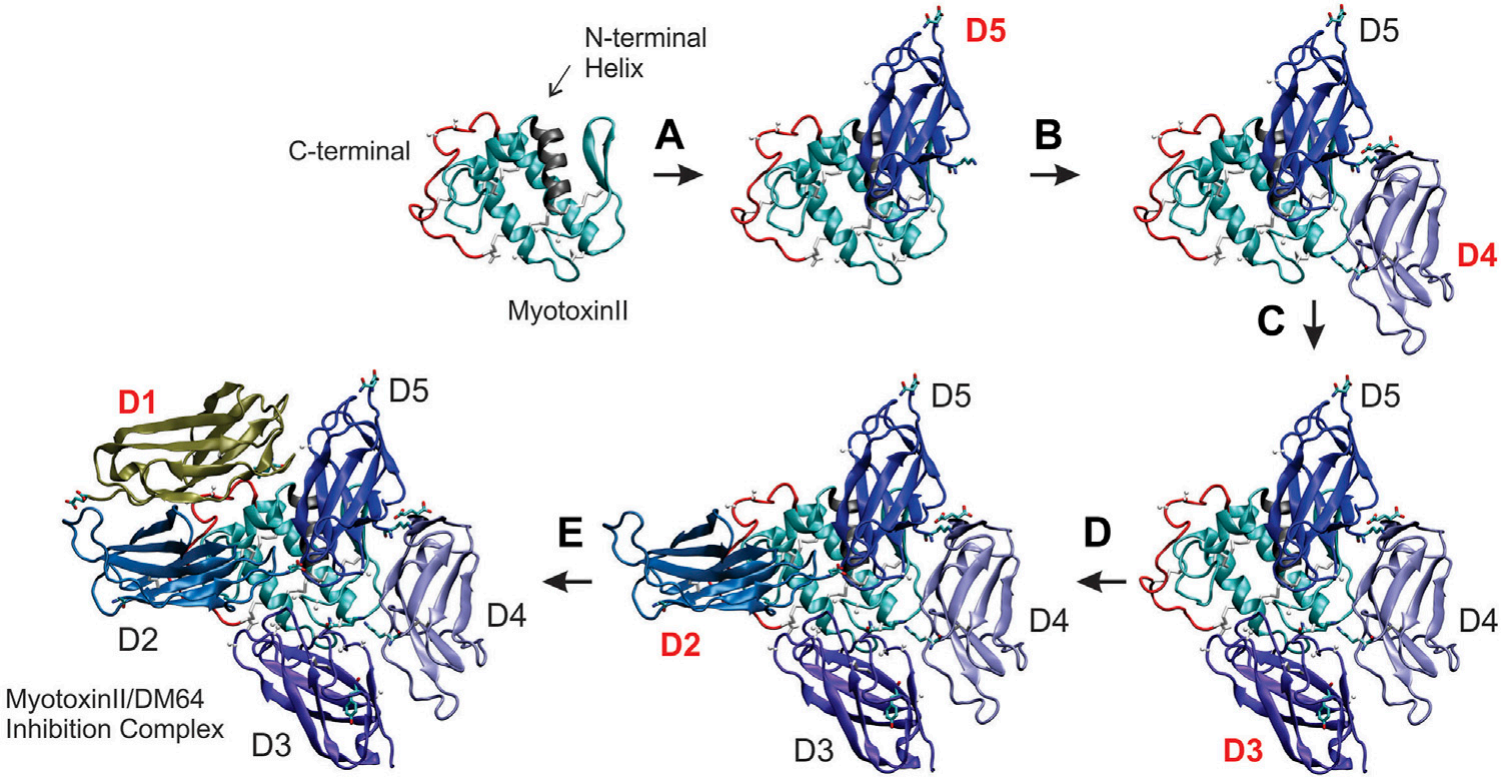
Building the structural model of DM64 docked with the crystal structure of myotoxin II. DM64 Ig-like domains were individually modeled and sequentially docked to myotoxin II (PDB ID 1CLP). Each docking step consisted of a global phase and a local phase when the interaction surface was refined. The first image in the upper left panel of the figure shows the ribbon representation of myotoxin II, with its highlighted N-terminal helix (residues 1 to 14; colored in gray) and the C-terminal region (residues 100 to 121; colored in red). **(A)** In the first docking step, the fifth Ig-like domain of DM64 (D5, colored in dark blue) was docked and interacted mainly with the N-terminal region of myotoxin II. **(B)** D4 (light blue) was docked at the frontal loop of myotoxin II, followed by **(C)** D3 (violet). **(D)** D2 (blue) was docked over the C-terminal region of myotoxin II, together with **(E)** D1 (tan).

Individual docking steps were sequentially shown in Figure 6 to better visualize DM64-myotoxin II interaction in the same spatial orientation. First, myotoxin II (1CLP, chain A) was represented by a ribbon cartoon, where the N-terminal helix (gray) and the C-terminal region (red) were highlighted to serve as reference points across the five docking steps. All Ig-like domains of DM64 were docked at the opposite side of the helices plane of myotoxin II, following the clockwise direction starting from domain D5 (Figure 6, step A).

**FIGURE 7:**
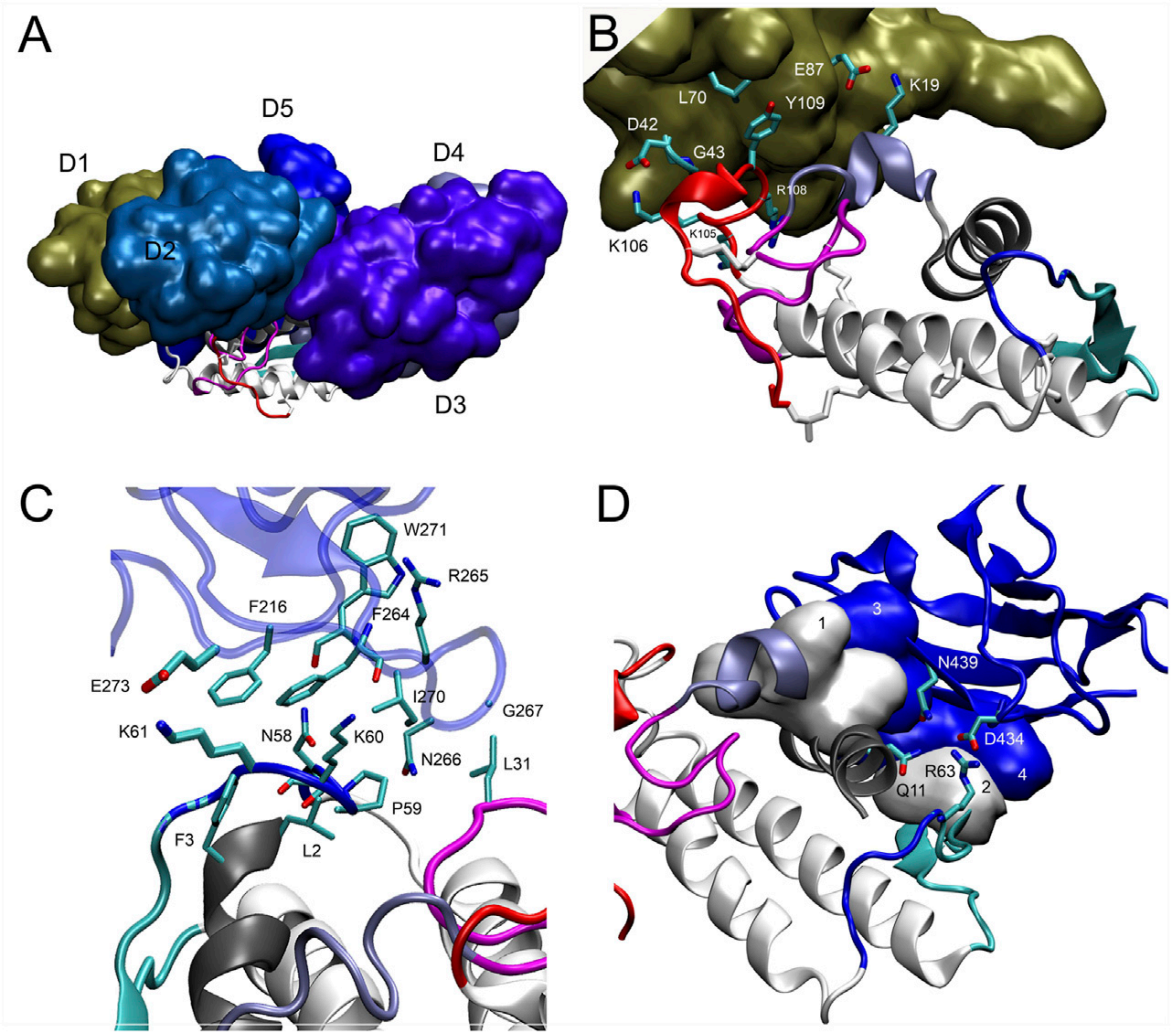
Interaction of myotoxin II and DM64 domains. **(A)** Lateral view of the complex model following the same color scheme of Figure 6. DM64 interacts throughout the curved interface of myotoxin II. Despite the complementarity of the surfaces, myotoxin II C-terminal peptide (colored in red) remains free. **(B)** Domain D1 (chain A) interacts with the MDoS residues K19, K105, and R108 of myotoxin II (chain F). The interaction surface encompasses hydrophobic contacts between Tyr109F/Leu70A and two long-lived hydrogen bonds between Lys106F/Asp42A and Lys19F/Glu87A. **(C)** Domain D3 (chain C) interacts with the frontal loop, preceding the beta-wing. The surface encompasses hydrophobic contacts between myotoxin II residues Phe3, Leu2, Leu31, Pro59, and D3 residues Phe216, Phe264, Arg265, Asn266, Gly267, and Ile270. **(D)** Domain D5 (chain E) interacts with the N-terminal helix (gray) and beta-wing (cyan) of myotoxin II (Chain F). Hydrophobic contacts at the myotoxin II interaction surface were represented as numbered white surfaces 1 (Leu10, Gly14 Lys15, and Asn16) and 2 (Trp68). On domain D5, the interaction region comprises blue surfaces 3 (Leu441 and Ile403) and 4 (His390, Val392, and His393). Additionally, several hydrogen bonds showing long-lived nature were detected on the interface, such as Arg63F-Asp434E and Gln11F-Asn439E.

In the final docking model of DM64-myotoxin II (Figure 7A), 26 out of 37 observed cross-linking restraints were within valid topological distances, corresponding to 70% overall validation. Roughly half of the cross-links connected DM64 and myotoxin II (14 validated links/18 observed links, 78% validation), whereas the remaining links were within DM64 (12 validations/19 observations, 63% validation). The main interactions between myotoxin II and D1, D3, and D5 from DM64 are illustrated in Figures 7B–D. As discussed below, these appear to be the most dominant regions for the interaction with myotoxin II.

### 3.7 SAXS Analysis

**FIGURE 8:**
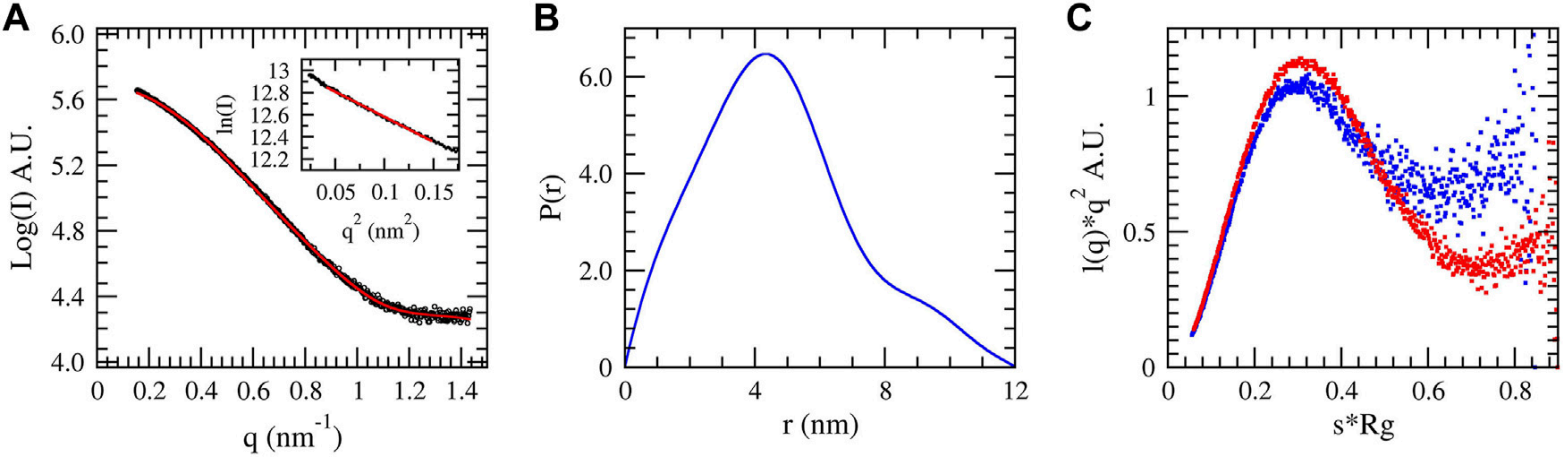
Small-angle X-ray scattering of the complex DM64-myotoxin II in solution. **(A)** Theoretical (red) and experimental (blue) scattering curves of the complex. The Guinier region is shown in the inset. **(B)** Pair distribution function P(r) of the complex. **(C)** Normalized Kratky plot obtained for DM64-myotoxin II complex (blue dots) and free DM64 (red dots).

SAXS data were collected for native DM64 in complex with myotoxin II to complement our structural analysis further. The scattering curve (Figure 8A) showed no evidence of aggregation of the sample. The linear Guinier plot (Figure 8A, inset) indicated that the system was essentially monodisperse. The distance distribution P(r) profile (Figure 8B) showed a non-gaussian shape, with a radius of gyration (Rg) of 3.71 nm and a maximum dimension (Dmax) of 12.02 nm. For comparison, the Rg value calculated for free DM64 was 3.52 nm. The normalized Kratky plot (Glatter and Kratky, 1982) for the complex was compatible with a folded protein presenting regions of flexibility (Figure 8C, blue dots). Interestingly, free DM64 showed similar compactness, with increased flexibility (Figure 8C, red dots).

### 3.8 Molecular Dynamics Simulation

Free molecular dynamics was used to assess the stability of the interaction between DM64 and myotoxin II. The PCA1 and PCA2 projections over 600 ns of MD simulation time of DM64-myotoxin II complex are shown in Supplementary Figure S11. The first 100 ns (grey dots) were considered an equilibrium period, whereas the black and blue dots represent the remaining 500 ns of MD simulation. From the equilibrium period to 300 ns, there was a transition in the conformational space (indicated by blue dots), followed by a stabilization in the PCA region (black dots farthest to the figure’s right).

**FIGURE 9:**
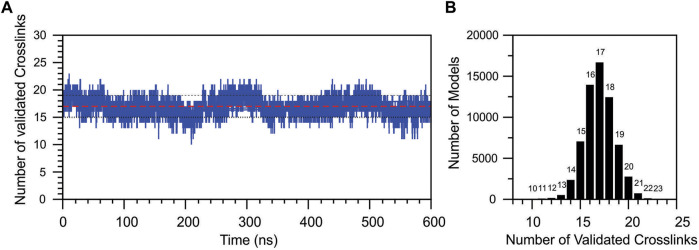
Topological validation of all cross-linking results during the molecular dynamics simulation of DM46-myotoxin II complex. **(A)** The number of validated cross-links was plotted as a function of the MD simulation time. The traced red line represents the average validation, and the black dotted lines represent the average fluctuation. **(B)** The total number of models produced in the MD simulation as a function of the number of validated cross-links.

To better understand the transition sampled in the PCA analysis, the topological validation of the identified cross-links was analyzed throughout the simulation time (Figure 9A). MD simulation started with the coordinates of the docking model of DM64-myotoxin II complex, initially containing 26 topologically validated cross-links. A cyclic fluctuation of validated cross-links was observed during the simulation, reflecting the breathing of the interaction in the time scale, originated by movements of the domains while interacting with myotoxin II and with the vicinal DM64 domains. The MD simulation showed a decrease in the total number of validated cross-links in the first nanoseconds; for 88.5% of the simulation time, a validation rate ranging from 15 to 19 validations was observed. The most populated state corresponded to models showing 17 topological cross-linking validations (Figure 9A, red line, and Figure 9B). A maximum of 23 validated cross-links was observed in a single model, and 22 validations were observed in only 52 models, both cases representing minor states sampled in the MD simulation.

**FIGURE 10:**
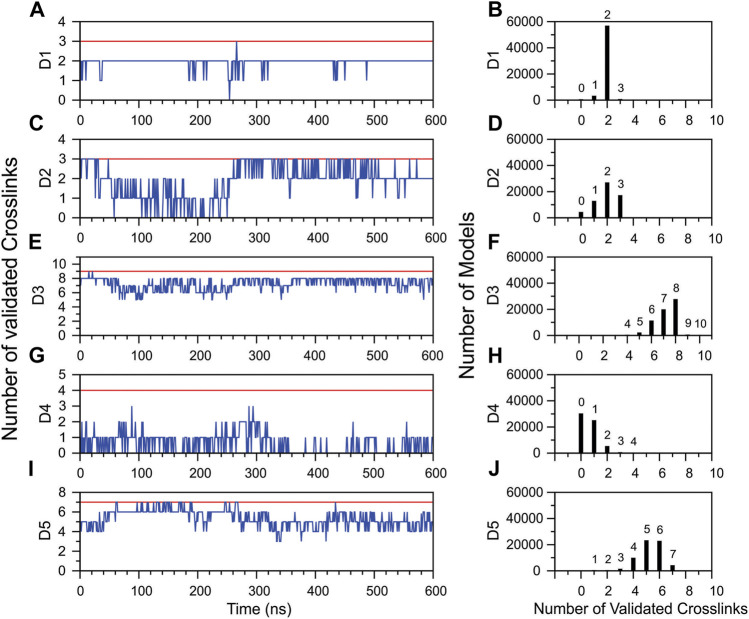
Topological validation of the cross-linking results within each Ig-like domain of DM64 during the molecular dynamics simulation of DM64-myotoxin II complex. On the left **(A,C,E,G,I)**, the blue line represents the average number of validations calculated over 100 frames as a function of simulation time. The red line indicates the maximum number of validated crosslinks identified in the DM64-myotoxin II docking model. On the right **(B,D,F,H,J)**, distribution of the total number of models produced in the MD simulation as a function of the number of validated cross-links.

To further investigate the reduction in the number of topologically validated cross-links during MD simulation, validations within each DM64 domain were individually analyzed. D1, D3, and D5 showed a relatively stable number of validations as a function of simulation time (Figures 10A,E,I). In the case of D1, of the three cross-links identified, two remained within topologically validated distances over 90.0% of the simulation time (Figure 10A). Almost all the ensemble models showed two validations, while the validation of all three cross-links was sampled for a minor group of 657 models (Figure 10B). In D3, seven and eight cross-links remained valid for 34.4 and 39.9% of the simulation time, respectively. Full validation of the cross-links was observed in very few models: 0.30% of the models showed nine validations, and 0.01% included ten links encompassing valid topological distances (Figure 10F). In D5, five validations remained constant for 38.5% of the simulation time, whereas six validations were observed for 35.0% of this interval. Full validation of the cross-links (all seven links) was observed during 6.9% of the simulation time (Figures 10I,J).

The glycosylated domains D2 and D4 were mainly responsible for decreasing the total validation rate (Figures 10D,H). D2 showed a validation drop in the first 250 ns of simulation, followed by a complete recovery immediately afterward (Figure 10C). Interestingly, the PCA analysis of MD simulations showed a transition in this time (Supplementary Figure S11, blue dots). On the other hand, D4 suffered a remarkable reduction in the number of validated cross-links at the beginning of the simulation, with no subsequent recovery to initial values. The number of validated links fluctuated between 0 and 1 for about half of the total simulation time (Figure 10G).

Hydrogen bond occurrences between individual DM64 domains and myotoxin II were monitored as a function of the simulation time (Supplementary Table S2 and Figure 7). Only the most prevalent hydrogen bonds (i.e., those with the most extended lifetimes) were used to infer stable interactions between protein surfaces. They were mainly observed in D5, where several significantly long-lived hydrogen bonds were detected, strikingly between Asn439 of D5-DM64 and Gln11 of myotoxin II. The lifetime of this hydrogen bond corresponded to 83% of the total simulation time. In D1, two long-lived hydrogen bonds were observed. They encompass myotoxin residues located at the toxic, membrane-destabilizing region (Asp42A-Lys106F) and the small helix (Glu87A-Lys19F). Finally, only short-lived hydrogen bonds were observed for D2, D3, and D4, with lifetimes corresponding to less than 10% of the total simulation time.

## 4 Discussion

*Bothrops* genus accounts for most snakebites in Latin America. Severe tissue damage is a major concern in bothropic envenomation, particularly in remote areas of the Amazon region. Even in cases not causing the victim’s death, these snakebites can frequently lead to the loss or disablement of limbs, with substantial physical, psychological, and socioeconomic impacts (Gutiérrez et al., 2006; Chippaux, 2017; Fan and Monteiro, 2018). Local toxicity includes edema, blistering, hemorrhage, dermonecrosis, and myonecrosis. Such pathological alterations are induced mainly by metalloendopeptidases and phospholipases A_2_ (Gutiérrez et al., 2017), which may exert synergistic toxic effects (Bustillo et al., 2012; Mora-Obando et al., 2014b). These venom proteins display fast-acting toxicity and wide antigenic variability, and their neutralization by conventional antivenom immunotherapy remains challenging (Gutiérrez et al., 1998).

The global burden of snake envenomation has been estimated at approximately six million disability-adjusted life years (DALYs), superior to most neglected tropical diseases (Habib et al., 2015). The development of new therapeutics is one of the urgent needs identified by the World Health Organization to reduce the suffering associated with snakebites (Williams D J et al., 2019). In line with this demand, the growing field of antivenom research has been devoted to studying the problem from various perspectives, including 1) the development of auxiliary diagnostic tests to identify the type/degree of envenomation; 2) the use of selected immunogens for improved antivenom efficacy; 3) the expansion of the treatment toolbox, including alternatives to be used in the field immediately after the bite (Laustsen et al., 2016; Williams H F et al., 2019; Knudsen et al., 2021).

From a therapeutic point of view, we argue that naturally occurring toxin inhibitors are valuable molecules. DM64, the only known myotoxin inhibitor of mammalian origin, binds to a wide variety of basic PLA_2_ (Rocha et al., 2017). Unlike the reptile PLI classes described in the literature (Ohkura et al., 1997; Campos et al., 2016), DM64 exerts its antitoxic activity without interfering with the catalytic activity of Asp49-PLA_2_ (Rocha et al., 2002). The present study sought to structurally explore the interaction between DM64 and a Lys49-PLA_2_ target, aiming to advance the knowledge about the mechanism of action of this naturally-occurring toxin inhibitor.

The primary structure of DM64 comprises 480 amino acid residues, adding up to 53.3 kDa. Its glycan moiety, corresponding to approximately 13 kDa, is responsible for the molecular heterogeneity observed in this molecule. The glycoprotein comprises five Ig-like domains (80–90 amino acid residues, including a central disulfide bond) connected by relatively conserved sequences of 12–13 amino acid residues (Supplementary Figure S4). The molecular masses here determined for DM64 by ESI-Q-TOF and AUC were in good agreement with previously determined values by MALDI-TOF MS (63.6 kDa) and SDS-PAGE under reducing conditions (66.5 kDa) (Rocha et al., 2002). It is worth mentioning that AUC results indicated a high anisotropy for DM64 (Table 1). Accordingly, protein glycosylation is consistent with higher frictional ratios (f/f_0_), resulting from increased hydration near hydrophilic sugar moieties. The presence of glycans could explain the anomalous behavior of DM64 previously observed by size exclusion chromatography (86–110 kDa) (Rocha et al., 2002), which had since then been primarily attributed to the dimerization of DM64 under native solution conditions. However, AUC can measure sedimentation and diffusion transport, providing a more reliable molar mass estimate that does not rely on reference standards or spherical shape assumptions. Therefore, it now seems clear that monomeric DM64 elutes earlier than expected in SEC due to glycosylation (León et al., 2012), leading to overestimating molar mass (Rocha et al., 2002). As already reported for DM64, an elevated anisotropy was also observed by AUC for the complex DM64-myotoxin II (Table 1).

The toxin used as a DM64 target in this study was myotoxin II from *Bothrops asper* venom, a well-known Lys49-PLA_2_ whose schematic structure is shown in Supplementary Figure S5 (Lomonte and Gutiérrez, 1989). Calcium-binding is prevented in myotoxin II due to a few amino acid modifications, mainly the critical substitution of Asp by Lys at position 49. Seven disulfide bonds stabilize myotoxin II structure, and substantial fluctuation in secondary or tertiary structure, even at the N-terminal and C-terminal regions, is not expected. The myotoxic activity of myotoxin II is mainly associated with its C-terminal sequence (105-KKYRYYLKPLCKK-117), as shown by studies with synthetic peptides. A combination of lysine and tyrosine residues forms a highly exposed cationic/hydrophobic region in the protein’s three-dimensional structure. The amphipathic character of this region is critical to the biological activity, and a triple tyrosine to tryptophan substitution in the C-terminal synthetic peptide of myotoxin II drastically enhanced its myotoxicity [reviewed in (Lomonte et al., 2003; Lomonte and Rangel, 2012)].

In myotoxin II’s apo form (PDB ID 1CLP), the N-terminal helix and the loop preceding the first β-strand of the beta-wing are implicated in the dimerization interface. This conventional dimer is stabilized by intermolecular hydrogen bonds involving Glu12, Trp68, and Lys71 [numbered Glu12, Trp77, and Lys80 in (Arni and Ward, 1995)]. On the other hand, the dimer adopted a novel conformation when myotoxin II was bound to the polyanionic compound suramin (Murakami et al., 2005). In this alternative dimer (PDB ID 1Y4L), the dimerization interface is formed by the hydrophobic surfaces surrounding the putative active site entry. Both oligomeric conformations are possible biological dimers, although bioinformatics analyses indicate that the alternative conformation is more stable in solution (Dos Santos et al., 2009; Fernandes et al., 2013).

A comprehensive toxic mechanism involving the alternative conformation of Lys49 myotoxins has been proposed based on structural data (Fernandes et al., 2013; Fernandes et al., 2014). Hydrophobic ligands (e.g., fatty acids binding in the hydrophobic channel of the toxin) would trigger a conformational shift that “activates” the dimer. The stabilization of this active conformation depends on interactions between residues in the putative Ca^2+^ binding loop and the C-terminal region. In this final conformation, the “membrane docking site” (MDoS, mainly formed by the cationic residues in the C-terminal region) and a cluster of critical nearby hydrophobic residues (MDiS, standing for “membrane disruption site”) in both monomers are perfectly positioned. The optimum interaction of the toxin MDoS with target anionic sites is followed by bilayer disruption through its MDiS. Consequently, there is a loss of control of ion influx across the membrane, irreversibly compromising the integrity of the muscle fiber (Gutiérrez and Ownby, 2003; Lomonte and Rangel, 2012). In myotoxin II from *Bothrops asper* venom, Lys19, Lys105, and Arg108 would form the putative MDoS. In contrast, MDiS would be formed by Leu111 and Leu114 [respectively residues 19, 115, 118, 121 and 124 in (Fernandes et al., 2014)], although a definitive identification to pinpoint the functionally critical residues of this toxin awaits experimental confirmation.

Because it is extremely sensitive (order of femtomoles) and relatively more tolerant to sample heterogeneity, mass spectrometry is gaining popularity in structural biology (Borch et al., 2005; Sinz, 2014). XL-MS involves stabilizing proteins/protein complexes with cross-linking agents, which covalently connect the side chains of specific residues. For the reaction to occur, these residues must be close enough in space for a sufficiently long time (Leitner et al., 2010). Cross-linking agents can vary according to the characteristic of the reactive groups (homo-/hetero-bifunctional), the reaction specificity (basic/acidic side chains or nonspecific/photoreactive), and the spacer chain length (0–20 Å). Additional options are also available in the cross-linker toolbox, such as cleavable, trifunctional, or isotopically labeled reagents (Sinz, 2003; Sinz, 2006).

After the cross-reaction, the proteins/protein complexes are enzymatically digested, and the cross-linked residues can be identified by high-resolution mass spectrometry. In this way, information about the maximum allowable distance between residue pairs is generated that increases structure prediction accuracy when used with computational modeling/docking tools (Sinz, 2014). This novel structural approach can provide valuable information regarding the spatial orientation of proteins and their connectivity. XL-MS results are often analyzed with data generated by complementary low-resolution structural methodologies (e.g., HDX-MS and SAXS) (Sali et al., 2015). This hybrid approach, known as integrative structural biology, has subsidized the formulation of consistent hypotheses connecting structure to biological function (Yu and Huang, 2018). Recently, XL-MS, HDX-MS, molecular modeling, protein-protein docking, and molecular dynamics simulations were successfully employed in the structural characterization of the first toxin-antitoxin complex. The study focused on the interaction between the endogenous circulating inhibitor BJ46a and the snake venom metalloendopeptidase jararhagin, both isolated from the South American snake *Bothrops jararaca* (Bastos et al., 2020).

A similar strategy was used here to advance our understanding of the molecular basis underlying the neutralizing activity of DM64 against a Lys49-PLA_2_ myotoxin. The use of a traditional lysine-reactive reagent allowed for the generation of valuable through-space distance information. Initially, free DM64 was modeled based on its whole amino acid sequence, and the homology model resulted in a structure enclosing the fundamental aspects of this protein class. Each Ig-like domain was structured in two β-sheets, composed of three and four β-strands, connected centrally by a disulfide bond. The loops connecting the β-strands in each sheet are the regions implicated in the interaction of the Ig-like domains with their molecular partners (Bork et al., 1994). The glycosylation sites were modeled exposed on the protein surface, allowing the addition of the glycan antennas. When cross-linking restraints were used to validate the final candidate model, distance violation rates indicated that the homology modeling succeeded well at the domain level (61.5% of intra-domain validation) (Supplementary Table S1B).

On the other hand, only 3.8% of the inter-domain cross-links were within the cross-linker maximum bound. In several cases, inter-domain cross-links spanning topological distances greater than 100 Å were measured (Supplementary Table S1B), clearly indicating that the global fold of the model did not correspond to the conformations experimentally sampled in the cross-linking experiment. The absence of suitable templates (i.e., encompassing consecutive Ig-like domains with high sequence identity) in the PDB data bank can explain the weak modeling performance. Even the recently published AlphaFold2 algorithm (Jumper et al., 2021) could not significantly improve the quality of the models compared to I-TASSER (not shown).

An alternative approach was then attempted to advance the structural understanding of the inhibitor and its interaction with myotoxin II. Distance constraints determined by cross-linking mass spectrometry were used to guide molecular modeling/docking strategies on the heterocomplex. The model was built based on observed cross-links connecting: 1) different domains of DM64; 2) DM64 domains and myotoxin II.

Structural determination methods are always influenced by time and conformational averaging effects (Markwick et al., 2008; Sun et al., 2019; Kulik et al., 2021). Such limitation also applies to structural methods using chemical cross-linking and high-resolution mass spectrometry. The identification of cross-linked peptides results from a chemical reaction with a diverse population of proteins in the solution, resulting in a dataset containing sub ensembles of cross-links that will never be validated simultaneously in a unique structural model. In docking analysis, we are chasing states of full validation of the experimental data. However, depending on the biological model, this scenario can never be reached. Therefore, free MD simulations were performed to sample the Ig-like domains’ dynamic behavior in the complex’s docked model. Given the low target–template sequence identity, the level of confidence of our model for the inhibitor is limited (if one considers the finer details of fold and surface features). Despite this, molecular dynamics simulation contributed to refining the results generated by the docking strategy, increasing our confidence in the final proposed model. Information on hydrogen bonding profiling was used with caution, being restricted to the most striking observations.

As the DM64-myotoxin II model was built after a series of docking steps, the choice of the initial docking partners was critical. D1 and D2 are glycosylated and showed low sequence coverages by mass spectrometry and the lowest number of identified cross-links. Most inter-domain cross-links were concentrated in D3 and D5. Interestingly, these domains were the source of the myotoxin-interacting peptides identified in the limited hydrolysis/affinity experiment. Although D3 showed the highest number of identified cross-links, D5 was most abundant in links connecting the inhibitor to the toxin, which was used as a reference point for the docking procedure. Hence, our first docking step focused on the interaction between D5 and myotoxin II.

D5 was docked to myotoxin II in the region of the N-terminal helix, the frontal loop, and the beta-wing (Supplementary Figure S6). Such interaction prediction encompassed the highest number of validated cross-links (77.8%) among all docked domains, indicating high interaction stability of this domain. An ensemble of anticorrelated cross-links was observed during the procedure: cross-links with N-terminal residues 1 and 15 in myotoxin II were never validated simultaneously with the remaining cross-links (Supplementary Table S1A). This result may indicate a second possibility for D5 interaction, leading to an alternative pathway for building the heterocomplex model structure. The alternative conformation represented a minor subset among 150,000 docking runs, and all attempts to proceed with docking the D4 domain resulted in low validation of inter-domain (D4-D5) cross-links.

The interaction between D5 and myotoxin II was analyzed in the MD simulation of DM64 and myotoxin II complex. Of seven validated cross-links in the initial docking model, between 5 and 6 links remained valid for 73.4% of the simulation time, indicating a highly stable interaction (Figures 10I,J). The partial loss of validated cross-links was expected and was attributed to structural relaxation during the model preparation phase (minimization, equilibration, and stabilization). D5-myotoxin II interaction surface also showed a higher number of long-lived hydrogen bonds, and they were verified in the N-terminal helix, the frontal loop, and the beta-wing (Supplementary Table S2E).

Residues involved in the most long-lived hydrogen bonds in DM64 (>40% of the total simulation time) were His405, Asn439, and Asp434. The latter is located in a loop (Val430-His436) connecting two β-strands, a region of Ig-like domains predicted to interact with molecular partners (Bork et al., 1994). The limited hydrolysis/affinity experiment identified two peptides from D5 binding to immobilized myotoxin II. The first one (residues 420–443) faces myotoxin II in the docked model of D5-myotoxin II (Supplementary Figure S6B, white surface). D5 seems critical for the interaction of the inhibitor with the toxin and the consequent neutralization of its toxic activity. It contributes to blocking the dimerization interface of the myotoxin (assuming a conventional dimeric conformation), thus preventing the oligomeric conformation that seems to be relevant for toxicity. Once bound to D5, myotoxin II would lose the ability to interact with the muscle membrane.

The second DM64 peptide fished by the myotoxin affinity column (residues 453–480) constitutes the C-terminal of the inhibitor. The docked model of DM64-myotoxin II did not show any contact region between this peptide and the toxin. Accordingly, no interaction could be observed in the MD, even when the simulation time was extended to 1 µs (not shown). The binding of the acidic 28 residues long peptide to the column may indicate that the docking model needs further refinement, although it can explain most of the experimental data obtained thus far. Another possibility involves alternative conformational states of the DM64-myotoxin II complex in solution, compatible with the observed interaction result. The remarkable sensitivity of mass spectrometry allows a most compelling exploration of the conformational space under analysis. This pattern is commonly observed in XL-MS experiments, where ambiguous data preclude all results’ simultaneous validation. Finally, we cannot rule out the possibility of nonspecific interaction between this C-terminal peptide and the column.

D4 was the first glycosylated (Asn355) domain added to the complex D5-myotoxin II (Supplementary Figure S7). The final docking model showed four validated cross-links (Supplementary Table S1A). However, the validation rate dropped early in the MD simulation and remained between zero and one valid link 50.0% of the time (Figures 10G,H). This behavior agrees with the hydrogen bond analysis, where long-lived hydrogen bonds could not be detected (Supplementary Table S2D). This scenario can be extrapolated to a dynamic interaction over time, including quick contact and detachment cycles from the myotoxin II surface.

Residues in domain D3 were engaged in 15 cross-links with D4, D5, and myotoxin II, nine of which could be validated in the final docking model (Supplementary Table S1A). Validated inter-protein cross-links indicate that D3 interacts with the toxin’s frontal loop (Supplementary Figure S8). MD simulation started with eight validated cross-links, and this number fluctuated between 6 and 8 for 95.0% of the simulation time, indicating a stable interaction (Figures 10E,F). Although only short-lived hydrogen bonds were observed in D3 (Supplementary Table S2C), they mainly involved DM64 residues Arg265 and Asn266 located in the D3 peptide 258–276, which was bound to myotoxin II in the limited hydrolysis/affinity experiment. A closer analysis of the interaction surface between D3-DM64 and myotoxin II also shows an important contribution of hydrophobic interactions.

We hypothesize that the interaction between the DM64 domains assumes acute angles, creating a structure in zigzag. In this conformation, while D5 interacts on the myotoxin II surface, D4 is pushed out in the direction of the solvent. Sequentially, another acute angle bend turns possible the return of D3 to the myotoxin II surface, probably allowing a higher interaction area with this domain. A similar structure was also described for murine paired immunoglobulin receptor B (PirB), leukocyte immunoglobulin-like receptor (LILR), and killer-cell immunoglobulin-like receptor (KIR) (Vlieg et al., 2019).

Alternative conformations upon the interaction of the third domain of DM64 may explain the more considerable discrepancy observed in the length of the transition region connecting the D3 and D4 domains (19.96 Å measured distance x 14.50 Å maximum expected distance). This result could also be derived from local conformational changes of DM64 upon binding, aiming to maximize the surface buried in the interaction, which would not be considered in molecular modeling. This possibility is supported by the consistent non-validation of cross-links between D3 and D4/D5 and the two non-validated crosslinks correlating D3 to myotoxin II C-terminal residues Lys105F and Lys106F (Supplementary Table S1A).

D2 has two glycosylation sites (Asn155 and Asn159) and established only one valid cross-link with the Lys61 residue of myotoxin II. Two additional valid links were observed with D3 (Supplementary Figure S9 and Supplementary Table S1A). A drop in the number of validated cross-links was observed after 60 ns and remained for 250 ns (Figures 10C,D). First, there was a shift from two to one validation and then from one to zero. After this period, the validation increased again and stabilized between 2 and 3 during the 300 ns of MD simulation. Domain D2 did not show hydrogen bonds with myotoxin II living in the “on” state longer than 0.5% of the MD simulation time (Supplementary Table S2B).

Finally, D1 was added to the docking model of D2D3D4D5-myotoxinII (Supplementary Figure S10). The experimental data acquired for this domain was limited, probably due to the high glycosylation content enclosed in the N-terminal domains D2 and D1. The final docking model shows three validated cross-links in D1, two connecting the inhibitor to Lys7 and Ser20 of myotoxin II (Supplementary Table S1A). The distance between domains D1 and D2 was about twice the expected maximum (23.03 × 10.67 Å), indicating that the model topology in this region is not sufficiently accurate. As MD simulation started, only two validated cross-links remained for 90.0% of the simulation time (Figures 10A,B).

Interestingly, the C-terminal peptide of the toxin (residues 99–121) was identified in the hydrolysate fraction bound to the DM64 affinity column. The docked model showed a significant interaction surface between this toxin region and the first two domains of DM64. In D1, long-lived hydrogen bonds were observed between residue Asp42 of the inhibitor and residue Lys106 of the toxin (Supplementary Table S2A). Despite the scarcity of experimental data on the first domain of DM64, the docking model, the MD simulation, and the hydrolysis experiment data support the hypothesis that D1 shows a relatively stable interaction with myotoxin-II. This result indicates that D1 likely blocks the MDoS site, impairing the anchoring of the toxin to the membrane.

Homology modeling of free DM64 always pointed to more elongated, U- or L-shaped structures. This shape is consistent with the non-gaussian distribution of pairwise distances derived from SAXS data and the high anisotropy observed by AUC. MD simulations showed that the connecting regions (10–12 residues) between the five Ig-like domains of DM64 are likely flexible. We hypothesize that the domains bend over myotoxin II, increasing the interaction surface area and the stability of the interaction. Accordingly, the normalized Kratky plot indicated a structured protein with pronounced flexibility when the inhibitor was free. After the binding, the complex showed nearly the same level of compactness, with a significant decrease in flexibility. The SAXS-derived Rg values for the complex (3.71 nm) and free DM64 (3.53 nm) were similar and correlated well with the hydrodynamic radii calculated by AUC (4.22 nm for the complex and 3.96 nm for DM64).

Crysol was used to quantify the goodness of fit between the experimental scattering data and those calculated from the MD simulation conformations. The correlation was weak when the final docking model was used as reference (Chi2 37.98, Rg = 2.84 nm). After 600 ns of MD simulation, the last coordinate showed a more relaxed protein structure, reducing the Chi2 value to 19.64 (Rg = 3.04 nm). Throughout the simulation time, the five most populated clusters showed Chi2 values ranging from 28.6 to 11.03, and minor represented conformations even reached 9.32. The best correlation was obtained by extending the simulation time to 1 µs (Chi2 = 4.40, Rg = 3.20 nm) (data not shown). This last structural model showed 16 validated cross-links, close to the mean validation rate observed in the MD analysis (17 validations, Figure 9). These results indicate that the MD cluster analysis can further improve the quality of the DM64-myotoxin II model. Although not ideal yet, the model is reasonably consistent with the experimental data and has supported valuable structural and functional predictions.

From a mechanistic point of view, DM64 inhibits myotoxin II from *Bothrops asper* venom in two presumed ways: 1) The fifth and the third domains interact with the toxin’s (conventional) dimerization surface. The binding precludes its quaternary structure assembly, which is relevant for myotoxicity; 2) The first domain directly interacts with the MDoS region, preventing the toxin’s anchoring to the muscle cell. Crystallographic studies with several small inhibitory ligands of PLA_2_-like toxins (e.g., varespladib, suramin) indicated the hydrophobic channel and the MDoS and MDis regions of the toxin as preferential functional targets (Salvador et al., 2019). Based on our topological data, it is difficult to speculate on the obstruction of the toxin hydrophobic channel by DM64. Previously, we have shown that DM64 inhibits the myotoxic activity of myotoxin I from *B. asper* yet cannot interfere with its catalytic activity (Rocha et al., 2002). This basic Asp49-PLA_2_ toxin and myotoxin II from *B. asper* show 61% sequence identity and conserved three-dimensional structures (α chains TM-score = 0.909) (Salvador et al., 2017). Thus, it seems reasonable to assume that the hydrophobic channel of myotoxin II likely does not participate in the mechanism of inhibition by DM64.

DM64 antitoxic protein scaffold has been naturally shaped by extensive trial and error experiments (Voss and Jansa, 2012). Therefore, unveiling the structural determinants of myotoxicity inhibition by DM64 can contribute insights into the rational design of therapeutic alternatives to treat snakebites. Notably, developing new peptide-based drugs as the first line of defense against venom-induced tissue damage is an attractive possibility (Erak et al., 2018). Compared to small molecules (<500 Da), peptide-based drugs are easier to produce and show higher potency and selectivity. Several alternatives to overcome possible limitations related to half-life, stability, solubility, and bioavailability are continuously being developed (Craik et al., 2013; Fosgerau and Hoffmann, 2015; Lau and Dunn, 2018). At least 60 peptide drugs are currently approved by the U.S. Food and Drug Administration (FDA) agency, and more than 600 are in (pre)clinical testing phases (Erak et al., 2018). The field of next-generation antivenom should not fail to explore innovative possibilities in such a blooming area.

## 5 Conclusion

Snake venoms are complex biological mixtures that induce a diversity of pathological effects. Yet, it seems possible to counteract their toxicity by inhibiting only a critical subset of toxins. For bothropic venoms, myotoxic PLA_2_s are priority targets. Using integrative structural biology, we have derived topological information on the complex made of a PLA_2_-like myotoxin and DM64. The latter is an endogenous protein that cross-neutralizes several homologous myotoxins. This study provides crucial insights towards understanding critical features of the inhibitor’s structure-function relationship.

## Data Availability

The datasets presented in this study can be found in online repositories. The names of the repository/repositories and accession number(s) can be found below: http://www.proteomexchange.org/, PXD028522.

## Supplementary Material

Supplementary Table S1 Cross-links identified by the software SIM-XL after applying the BS^3^ strategy on DM64 and myotoxin II complex. The tables list all high-confidence cross-links and the analysis of Euclidean and topological distances (Cβ-Cβ atoms) calculated with the software TopoLink.

Supplementary Table S2 Free MD hydrogen bond lifetimes in the interaction surfaces between each Ig-like domain of DM64 and myotoxin II.

Supplementary Figure S1 Analysis of complex formation between DM64 cross-linked with BS^3^ and native myotoxin II. The binding property of cross-linked DM64 toward native myotoxin II was monitored on a non-denaturing 12% T gel stained with Coomassie blue R250. Lane 1: DM64 (native control); lane 2: DM64 cross-linked with BS^3^ (1:2,800 mol/mol); lane 3: DM64 + BS^3^ (1:2,000 mol/mol); lane 4: DM64 + BS^3^ (1:1,000 mol/mol); lane 5: DM64 (native control) + mtx-II; lane 6: [DM64 + BS^3^ (1:2,800 mol/mol)] + mtx-II; lane 7: [DM64 + BS^3^ (1:2,000 mol/mol)] + mtx-II; lane 8: [DM64 + BS^3^ (1:1,000 mol/mol)] + mtx-II; lane 9: mtx-II.

Supplementary Figure S2 Stabilization of the complex made by the antitoxin DM64 and myotoxin II using different concentrations of BS^3^. **(A)**: Electrophoresis under non-denaturing conditions; **(B)**: Electrophoresis in the presence of SDS and DTT. All samples were run on 12% T gels stained with silver nitrate. MM: molecular mass markers; lane 1: DM64 (native control); lane 2: DM64 cross-linked with BS^3^ (1:2,800 mol/mol); lane 3: mtx-II (native control); lane 4: mtx-II + BS^3^ (1:2,800 mol/mol); lane 5: (DM64 + mtx-II) + BS^3^ (1:2,800 mol/mol); lane 6: (DM64 + mtx-II) + BS^3^ (1:2,000 mol/mol); lane 7: (DM64 + mtx-II) + BS^3^ (1:1,000 mol/mol); lane 8: (DM64 + mtx-II) + BS^3^ (1:500 mol/mol).

Supplementary Figure S3 Myotoxin II cross-linked with BS^3^. Ribbon diagram of myotoxin II (1CLP, chain A) showing the topologically validated cross-linking connections colored in green, the N-terminal α-helix (residues 1 to 15, colored in gray), the analogous region for calcium coordination site in catalytically active enzymes (residues 28 to 33, colored in magenta) and the myotoxic peptide (residues 100 to 121, colored in red). The active site residues His47, Tyr51, and Asp89, were represented in sticks. The cross-linking reaction was performed on myotoxin II in complex with DM64.

Supplementary Figure S4 Primary structure of the inhibitor DM64. A sequence of five amino acid residues was highlighted at each Ig-like domain’s start and end (yellow boxes). Four conserved regions formed by 12-13 amino acid residues (cyan boxes) connected sequential Ig-like domains. Except for the underlined sequences, the connection regions were included in the modeling of the respective domain, as detailed in Table 2.

Supplementary Figure S5 Ribbon representation of the crystal structure of myotoxin II (1CLP, chain A) with the main structural elements highlighted in color. Myotoxin II structure is built over a helix plane formed by two long antiparallel α-helices (white). The N-terminal is structured in an α-helix (residues 1-14, gray) bent to create a small helix (residues 16 to 24, blue). The sequence analogous to the calcium coordination loop in catalytically active phospholipases A_2_ was colored magenta (residues 25 to 39). Sequentially, residues from 40 to 57 form the first long α-helix (white). The region from residues 58 to 73 corresponds to the frontal loop (blue) that connects to a short antiparallel β-sheet (residues 63-86, cyan) named beta-wing, followed by the second long α-helix (89-108, white). Finally, the myotoxic C-terminal peptide extends from residue 109 to 133 (red). The numbers in parenthesis indicate the following disulfide bonds (yellow): (1) Cys50-Cys88, (2) Cys43-Cys95, (3) Cys57-Cys81, (4) Cys28-Cys44, (5) Cys49-Cys121, (6) Cys26-Cys115, and (7) Cys75-Cys86.

Supplementary Figure S6 Ribbon representation of the fifth Ig-like domain of DM64 docked with the crystal structure of myotoxin II. **(A)** D5 (blue) interacting with myotoxin II (cyan), with the C-terminal myotoxic peptide (residues 100 to 120) colored in red and the N-terminal helix (residues 1 to 14) highlighted in grey. All five validated cross-links involving Lys443 in D5 and myotoxin II are indicated with dotted green lines (topological distances). The relative position of both proteins in the complex was fixed by cross-link Ser407E-Lys69F, which restricted the movements of the interaction surface. **(B)** The same as in panel A, with the peptide 420-443 from DM64 interacting with myotoxin II, as shown by the limited hydrolysis/affinity experiment, represented in white surface.

Supplementary Figure S7 Ribbon representation of the fourth Ig-like domain of DM64 docked with D5Myotoxin II. D4 (light blue) interacts with D5 (dark blue) and myotoxin II (cyan), with the C-terminal myotoxic peptide (residues 100 to 120) colored in red and the N-terminal helix (residues 1 to 14) highlighted in grey. Myotoxin residue Lys61 lies in front of the N-terminal helix and is involved in a cross-link with Lys289D, stabilizing D4 and myotoxin II interaction. All validated cross-links following this second docking step are indicated with dotted green lines (topological distances).

Supplementary Figure S8 Ribbon representation of the third Ig-like domain of DM64 docked with D4D5Myotoxin II. DM64 domain D3 (violet) interacting with D4 (light blue), D5 (dark blue), and myotoxin II (cyan). Residues Lys52, Lys60, and Lys 61 are in the loop connecting the two helices of the myotoxin II structure.

Supplementary Figure S9 Docking the second Ig-like domain of DM64 with D3D4D5Myotoxin II. Residue Lys117 in D2 (navy blue cartoon) was cross-linked to residues Lys217 and Lys241in D3 (violet cartoon), and myotoxin II (cyan cartoon), through residue Lys61, located in the loop connecting the two long helices. Domains D5 (dark blue) and D4 (light blue) were represented as surfaces for clarity.

Supplementary Figure S10 Docking the first Ig-like domain of DM64 with D2D4D5Myotoxin II. D1 (tan cartoon) is interacting with D2 (navy blue surface), D3 (violet surface), D4 (light blue surface), D5 (dark blue surface), and myotoxin II (cyan cartoon). Residue Ser84 connects Myo-II residues Lys7 and Ser20 located at the N-terminal helix. Leu1 residue at D1 N-terminal connects residue Ly443 at the C-terminal portion of D5. In addition, the glycosylation site Asn22 is exposed on the D1 surface.

Supplementary Figure S11 Principal Component Analysis (PCA) of molecular dynamics simulations. The equilibrium period (0–100 ns) was represented by gray dots, whereas black dots represent the production phase. A shift was observed in the time window up to 300 ns (blue dots).
