# Supplementary figures and images for: Molecular Architecture of the Antiophidic Protein DM64 and its Binding Specificity to Myotoxin II From Bothrops asper Venom
Source: Front Mol Biosci. 2022 Jan 27;8:787368. doi: 10.3389/fmolb.2021.787368 (PMC8830425; doi:10.3389/fmolb.2021.787368)

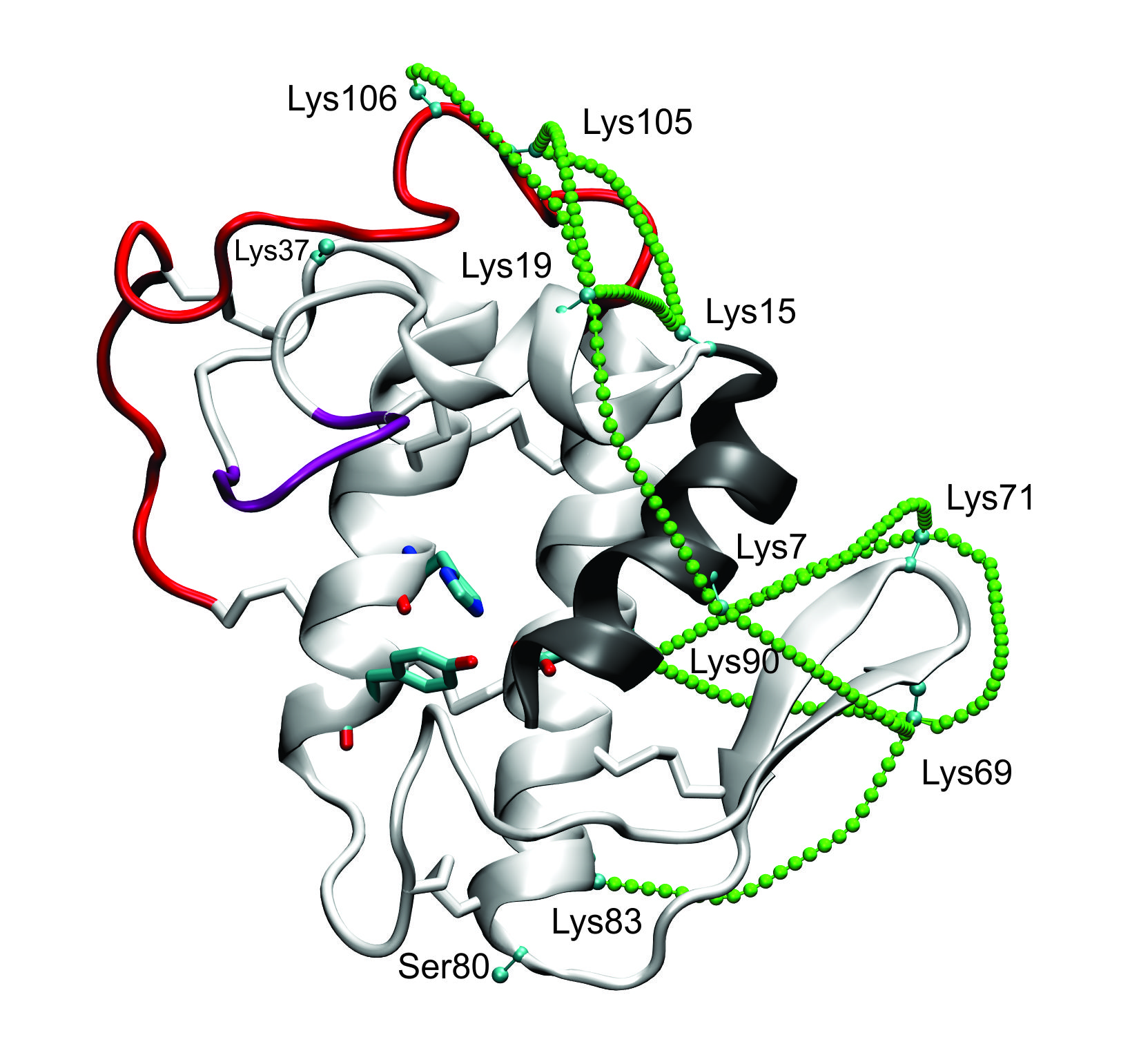

Supplement: Supplementary file 2 [file Image3.JPEG]

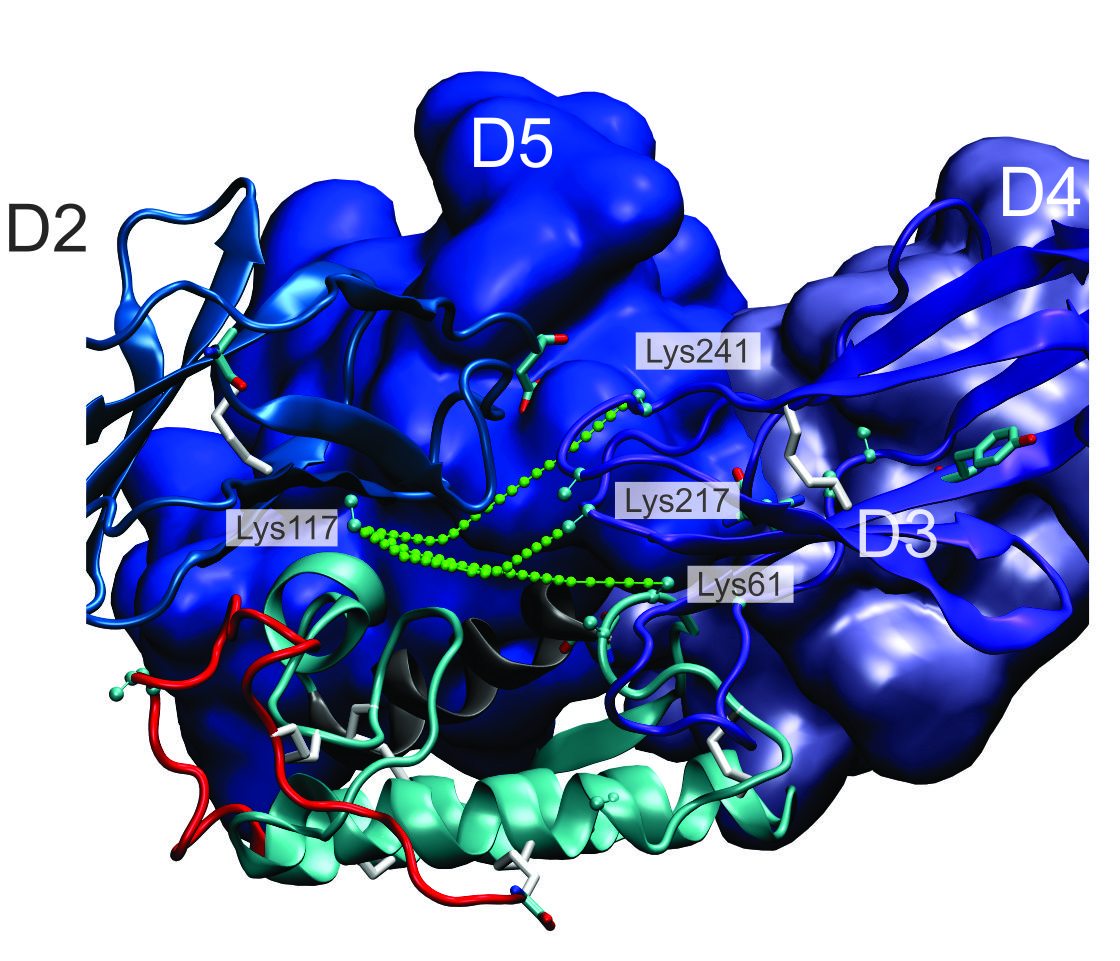

Supplement: Supplementary file 3 [file Image9.JPEG]

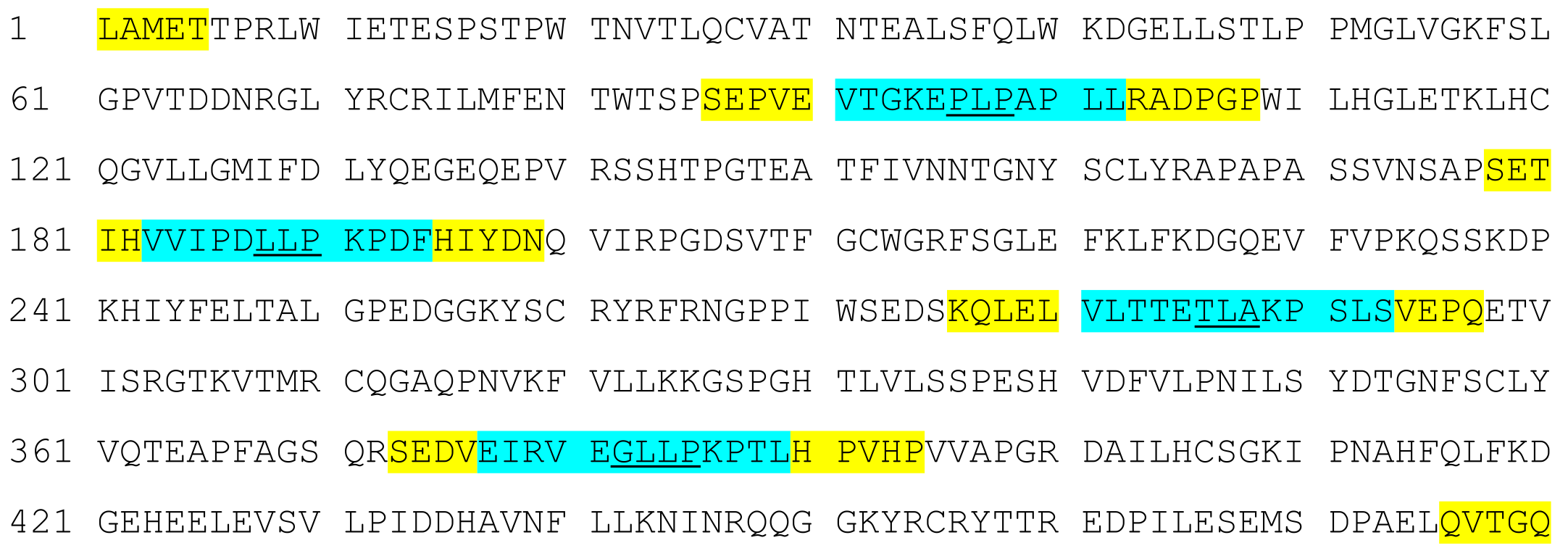

Supplement: Supplementary file 4 [file Image4.TIF]

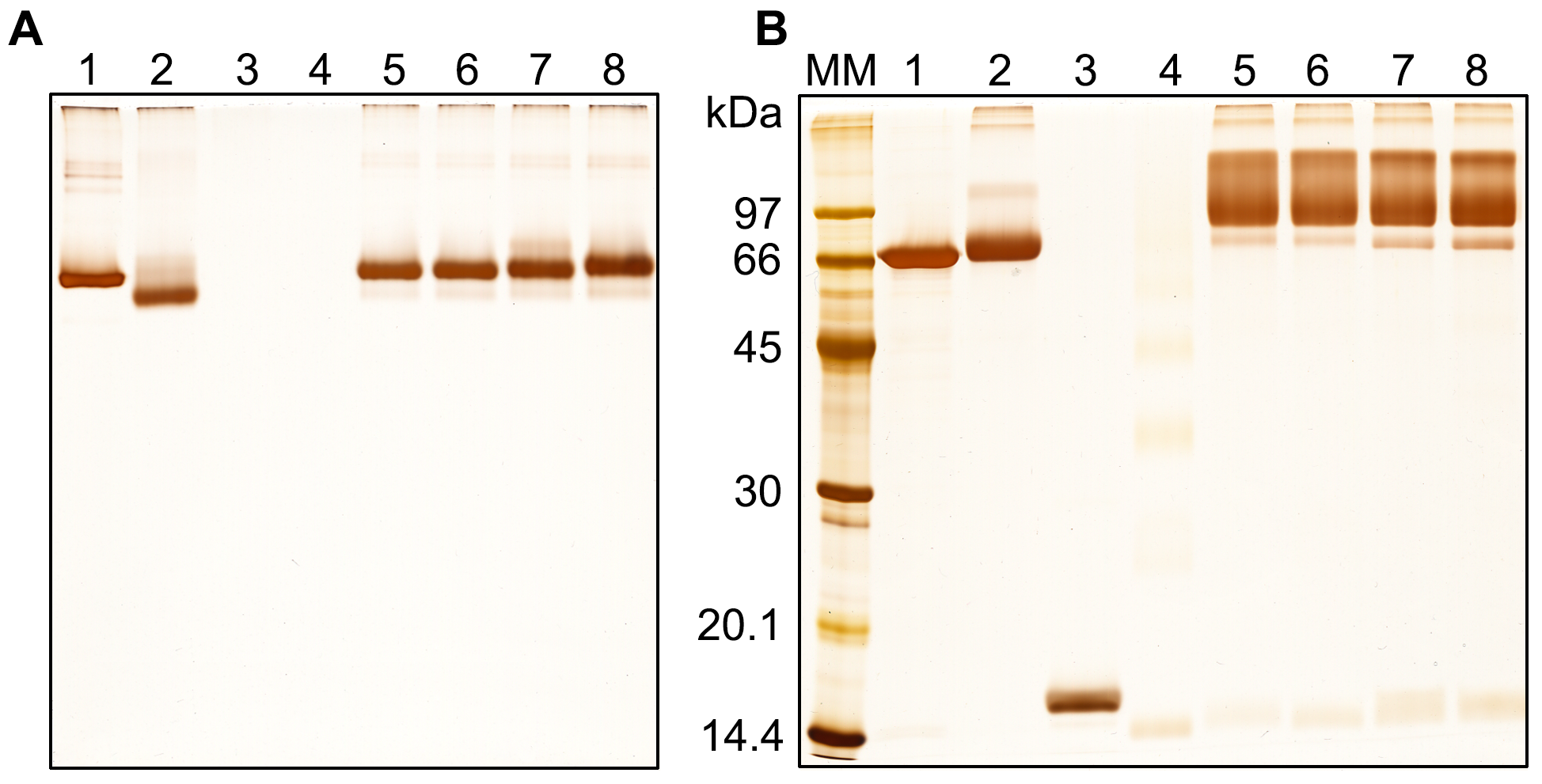

Supplement: Supplementary file 5 [file Image2.TIF]

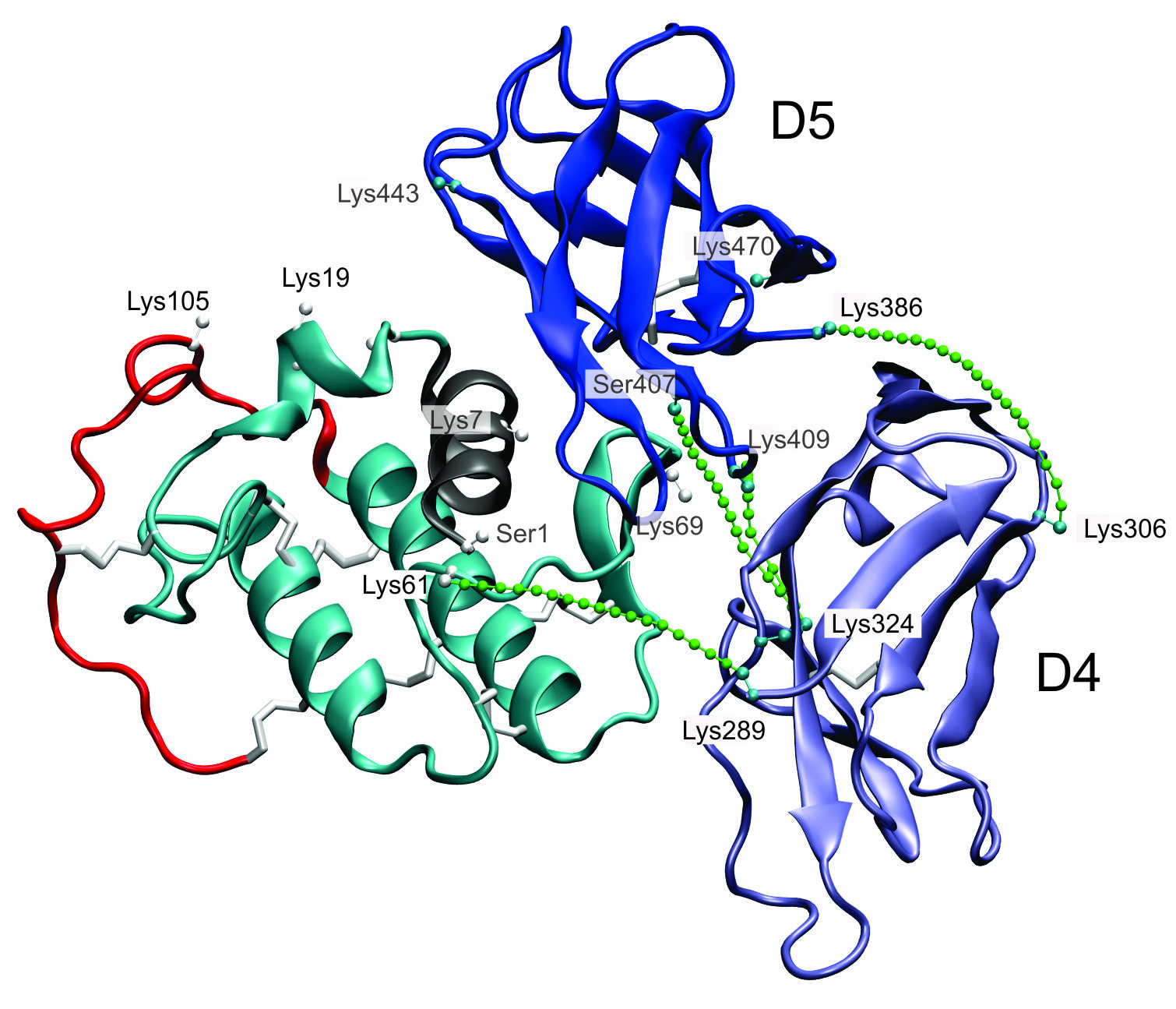

Supplement: Supplementary file 6 [file Image7.JPEG]

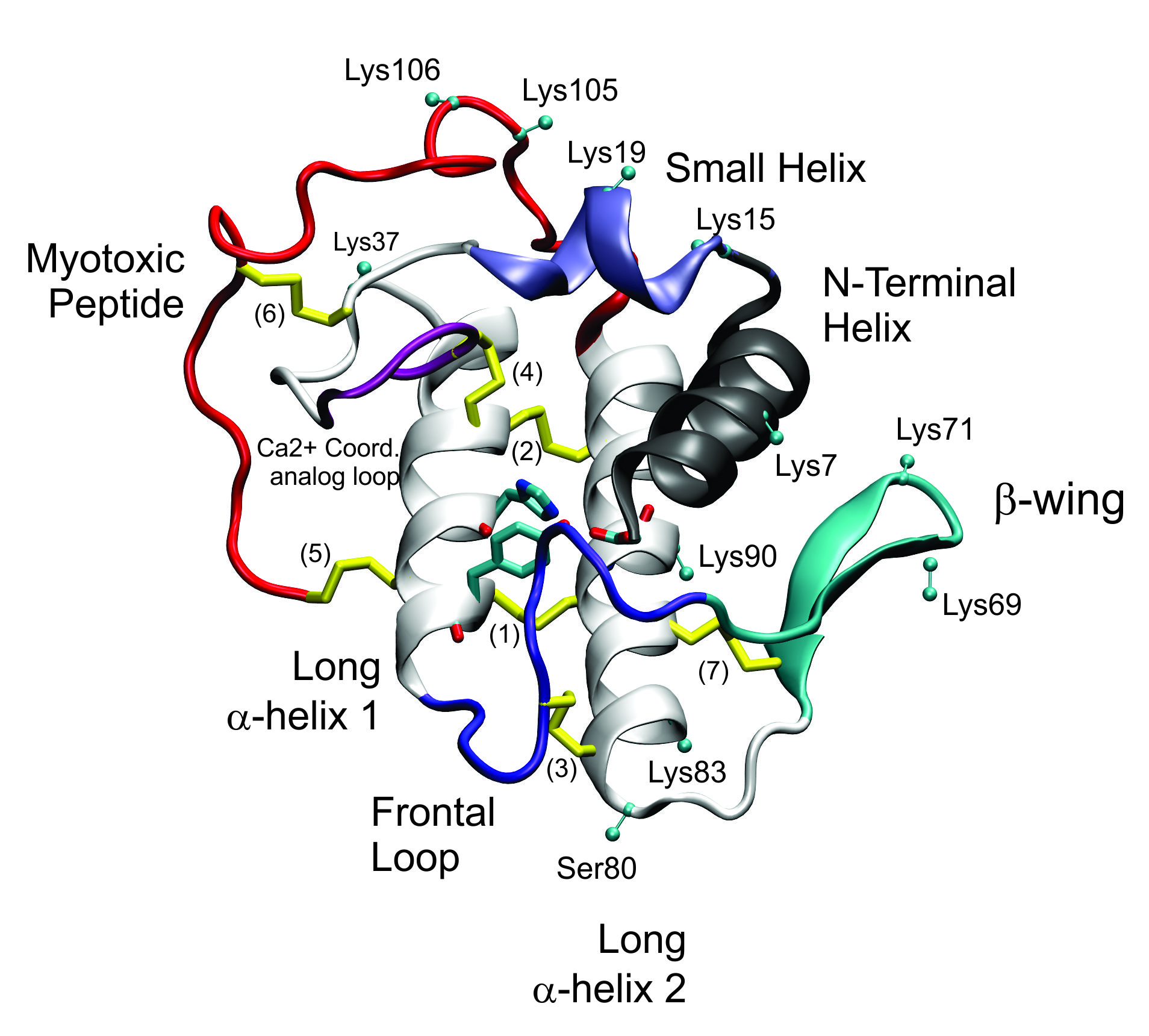

Supplement: Supplementary file 7 [file Image5.JPEG]

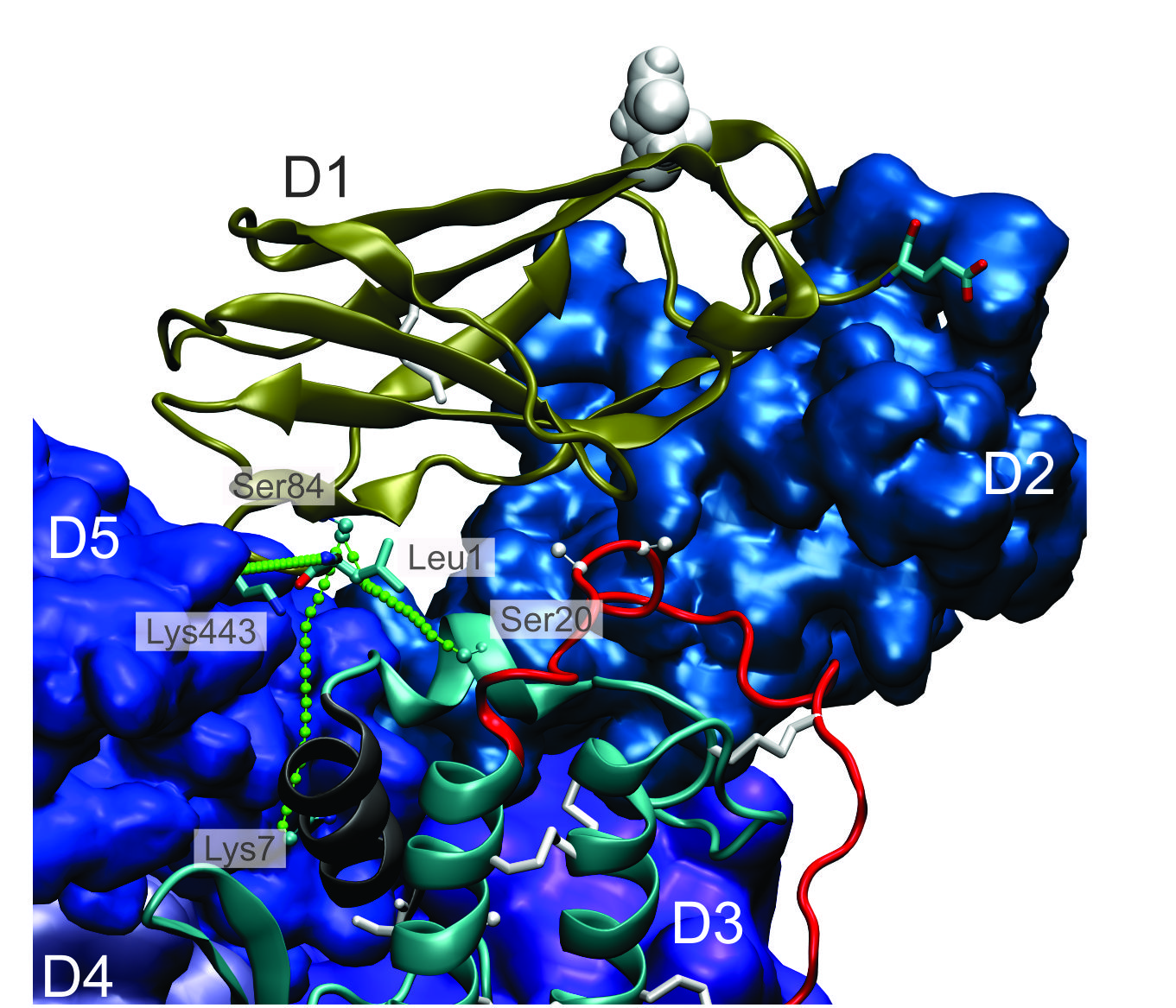

Supplement: Supplementary file 8 [file Image10.JPEG]

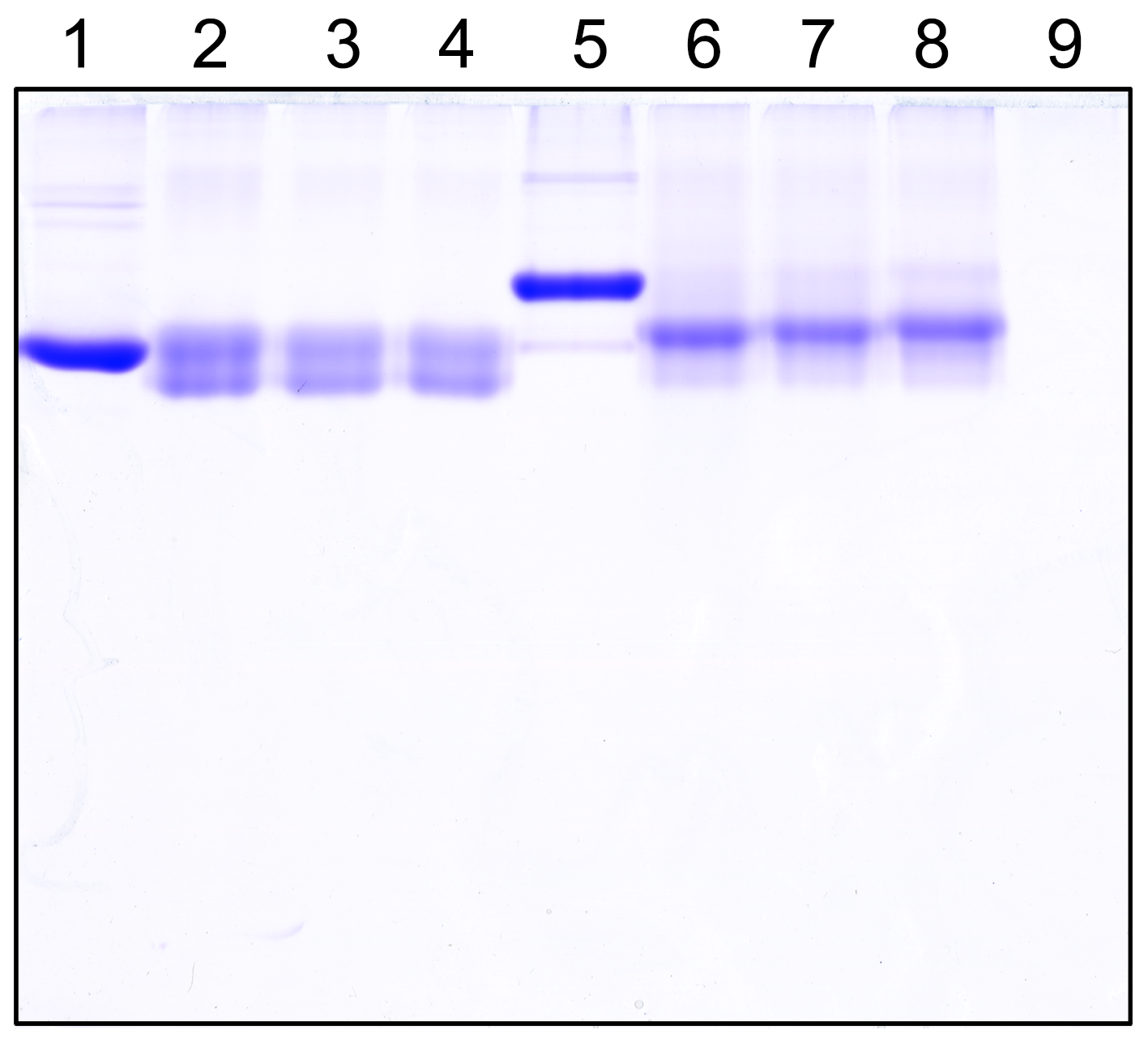

Supplement: Supplementary file 9 [file Image1.TIF]

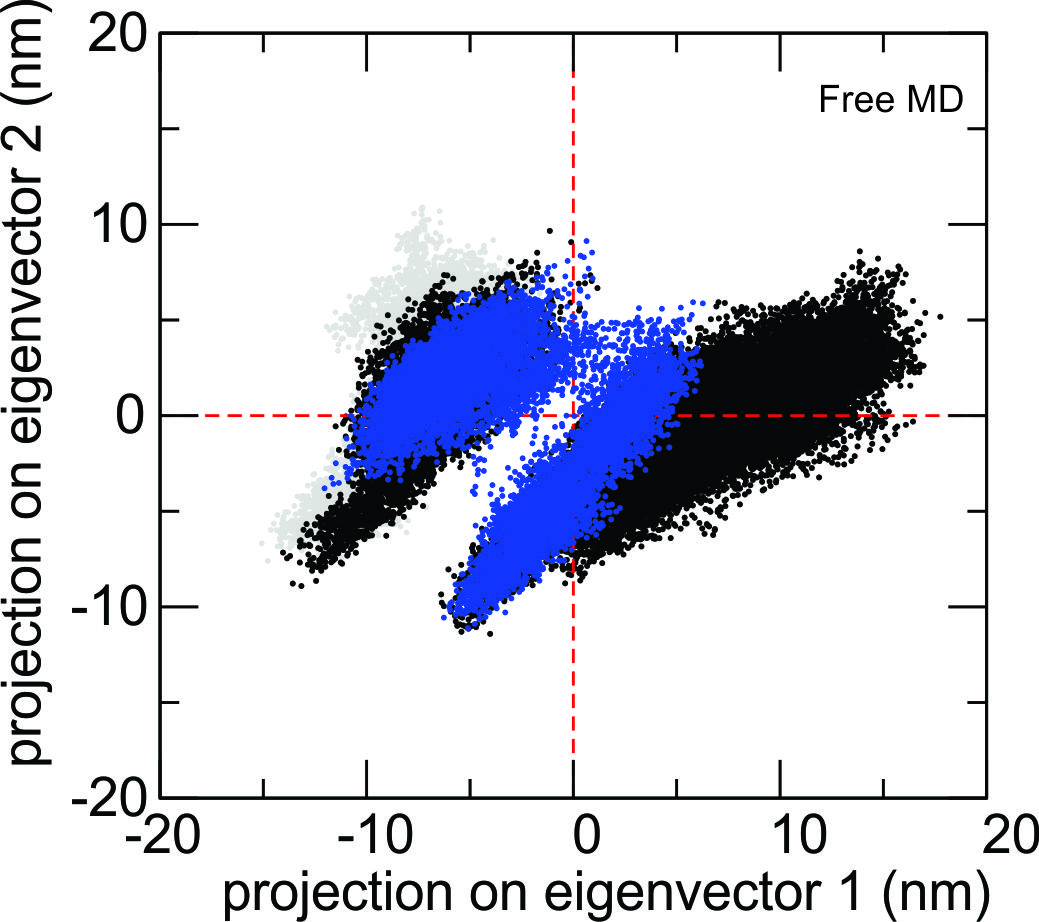

Supplement: Supplementary file 10 [file Image11.JPEG]

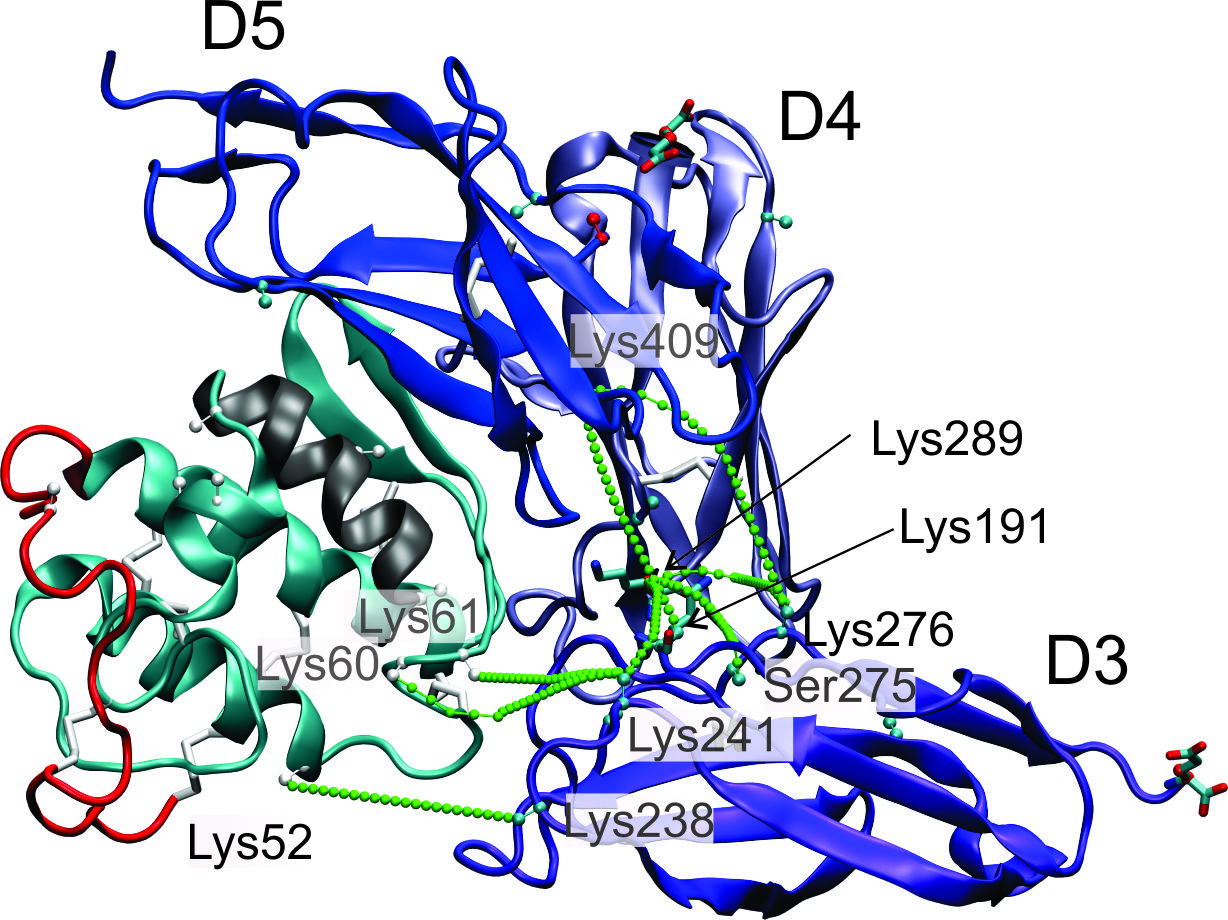

Supplement: Supplementary file 12 [file Image8.JPEG]

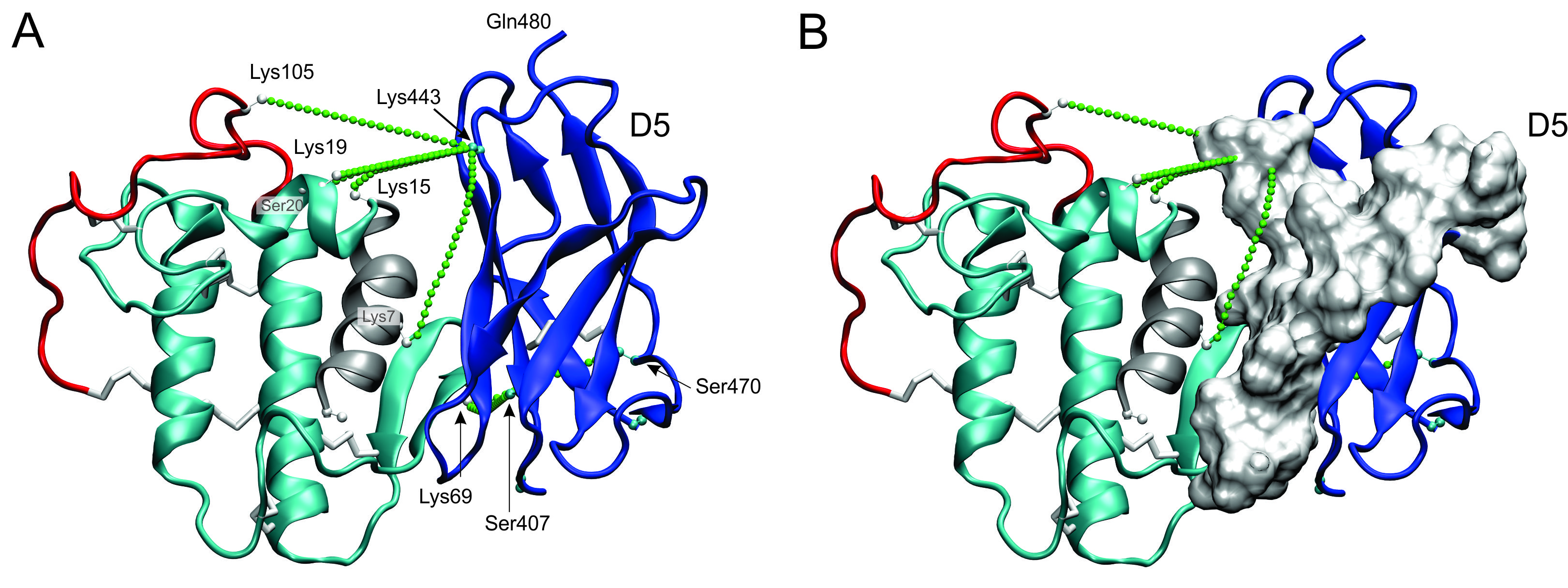

Supplement: Supplementary file 13 [file Image6.JPEG]
